# Atomic Motif Recognition in (Bio)Polymers: Benchmarks From the Protein Data Bank

**DOI:** 10.3389/fmolb.2019.00024

**Published:** 2019-04-18

**Authors:** Benjamin A. Helfrecht, Piero Gasparotto, Federico Giberti, Michele Ceriotti

**Affiliations:** Laboratory of Computational Science and Modeling, Institute of Materials, École Polytechnique Fédérale de Lausanne, Lausanne, Switzerland

**Keywords:** atomistic and molecular simulation, machine learning, biomolecules, molecular motifs, hydrogen bonds, secondary structure

## Abstract

Rationalizing the structure and structure–property relations for complex materials such as polymers or biomolecules relies heavily on the identification of local atomic motifs, e.g., hydrogen bonds and secondary structure patterns, that are seen as building blocks of more complex supramolecular and mesoscopic structures. Over the past few decades, several automated procedures have been developed to identify these motifs in proteins given the atomic structure. Being based on a very precise understanding of the specific interactions, these heuristic criteria formulate the question in a way that implies the answer, by defining a list of motifs based on those that are known to be naturally occurring. This makes them less likely to identify unexpected phenomena, such as the occurrence of recurrent motifs in disordered segments of proteins, and less suitable to be applied to different polymers whose structure is not driven by hydrogen bonds, or even to polypeptides when appearing in unusual, non-biological conditions. Here we discuss how unsupervised machine learning schemes can be used to recognize patterns based exclusively on the frequency with which different motifs occur, taking high-resolution structures from the Protein Data Bank as benchmarks. We first discuss the application of a density-based motif recognition scheme in combination with traditional representations of protein structure (namely, interatomic distances and backbone dihedrals). Then, we proceed one step further toward an entirely unbiased scheme by using as input a structural representation based on the atomic density and by employing supervised classification to objectively assess the role played by the representation in determining the nature of atomic-scale patterns.

## 1. Introduction

Macromolecules are characterized by their capability of folding and assembling into hierarchical structures, which is a crucial element in their activity and stability. Understanding the structure of a macromolecule is thus a key step in discerning its function. Proteins are the archetypal example of complex molecular machines designed to perform unique and well-defined operations. Many polypeptides exhibit distinct secondary and tertiary structures in their native state, which are often used to explain their behavior. However, understanding and characterizing the structure of a macromolecule, even in the case of small proteins, can be rather difficult.

The structural description of a non-rigid molecule with many degrees of freedom relies on the identification of motifs, which can be used to classify their three-dimensional structure (e.g., an alpha-helix or beta-sheet in the case of a protein). The most common motifs that characterize these kinds of structures are intramolecular hydrogen bonds, such as those present in polysaccharides, as well as distinct dihedral angle patterns that are assumed by the backbone of a protein. Much work has been dedicated to understanding and classifying hydrogen bonds, ultimately producing several geometric criteria (e.g., distances and angles between donors and acceptors) as well as energetic criteria, to identify their presence or absence (Rahman and Stillinger, 1971; Brown, 1976; Mezei and Beveridge, 1981; Baker and Hubbard, 1984; Luzar and Chandler, 1993; McDonald and Thornton, 1994; Luzar and Chandler, 1996; Xu et al., 1997; Desiraju and Steiner, 2001; Arunan et al., 2011; Jeffrey and Saenger, 2012). Likewise, tabulating the different backbone dihedral angles exhibited by a macromolecule produces the so-called Ramachandran plot (Ramachandran et al., 1963), which finds widespread use in chemistry, biology, and biophysics to aid in the identification of protein secondary structure (Frishman and Argos, 1995).

There are several examples where this motif-based rationale was successfully employed to identify the secondary structure of proteins; the DSSP (Kabsch and Sander, 1983) and STRIDE (Frishman and Argos, 1995) algorithms are two notable examples. However, the identification of structural motifs in proteins is often based on a combination of human intuition and—sometimes generous—approximations, and may not be unique or readily applicable to different macromolecules. Moreover, the motif definitions are typically based on assessments of specific structures or, in the case of the hydrogen bond, focus solely on a single subset of the atomic species that may be involved.

In this context, a statistical framework capable of automatically identifying structural motifs that is free of energy approximations and relies on system-agnostic definitions would be advantageous. Having a purely data-driven definition of various motifs would be particularly useful in the field of bioinformatics, where they are used for structure prediction or the development of scoring functions for processes like protein-ligand docking. For example, Rosetta, one of the most well-known energy functions, has been developed to predict the structure of a protein given its amino acid sequence and local structural features such as dihedral angles (Simons et al., 1997, 1999).

Another example where purely data-driven definitions would be advantageous is in secondary structure classification. While several methods exist to classify protein secondary structure (Kabsch and Sander, 1983; Frishman and Argos, 1995, 1996; Jones, 1999; Cuff and Barton, 2000; Andersend et al., 2002; Martin et al., 2005; Nagy and Oostenbrink, 2014; Haghighi et al., 2016), these methods rely on amino acid sequences, hydrogen bonding energies, geometrical criteria, or some combination thereof. Machine learning techniques (Muggleton et al., 1992), and neural networks in particular (Holley and Karplus, 1989; Rost and Sander, 1993a,b; Jones, 1999; Cuff and Barton, 2000; Akkaladevi et al., 2004; Wood and Hirst, 2005; Rashid et al., 2016; Zhang et al., 2018) have also been used to classify the secondary structure of a protein based on a variety of features. Others have developed schemes to classify conformational patterns and secondary structure using dihedral angles alone (Hollingsworth et al., 2012; Nagy and Oostenbrink, 2014), but there remains a lack of a truly agnostic method for classifying (and predicting) secondary structures.

In this work, we illustrate how it is possible to use machine learning to obtain a statistical definition of atomic-scale motifs based on a data-driven analysis. Given a descriptor of the atomistic environments, we construct a probability density representing its occurrence in a given dataset. Then, using the Probabilistic Analysis of Molecular Motifs (PAMM) algorithm (Gasparotto and Ceriotti, 2014; Gasparotto et al., 2018), which casts the probability density into a Gaussian mixture model (GMM), we find the most probable motifs in the distribution. To create the density distribution we have used two different approaches: one using classical geometric descriptors such as interatomic distances and dihedral angles, and a more agnostic scheme that uses the Smooth Overlap of Atomic Positions (SOAP) framework (Bartók et al., 2013; Bartók and Csányi, 2015; De et al., 2016) as the input representation. The motif fingerprints obtained in this way have a general definition and are transferable between different systems. To illustrate this point, rather than selecting proteins of a given family or with small variations in the sequence, we have used entries from the Research Collaboratory for Structural Bioinformatics Protein Data Bank (RCSB PDB) (Berman et al., 2000). The motifs obtained from PAMM were compared to a more “traditional” geometric definition of a hydrogen bond and to DSSP- and STRIDE-assigned secondary structures to assess their similarity. Furthermore, by comparing the fidelity of the unsupervised classification given by PAMM with that of a supervised scheme, we can assess whether classification errors stem from an incomplete representation or are a manifestation of the arbitrary nature of heuristic methods.

## 2. Methods

The methods we used to represent structures and identify molecular motifs have been already discussed elsewhere. We used the PAMM scheme (Gasparotto and Ceriotti, 2014; Gasparotto et al., 2018) to identify modes in the probability distribution of atomic patterns. The PAMM algorithm takes as input a series of vectors representing local environments (distances, angles or more generic density-based representations such as SOAP feature vectors Bartók et al., 2013; De et al., 2016), performs a kernel density estimation on a sparse grid obtained by subsampling the input data, and performs a density-based clustering to identify local maxima in the estimate of the probability distribution. Finally, each cluster is represented as a Gaussian mode, which makes it possible to define probabilistic motifs identifiers (PMIs), structural indicators taking a value between zero and one that represent the degree of confidence by which a new local structure can be assigned to each of the clusters. In what follows we only summarize the aspects that are relevant to this specific application, explaining in detail the preparation of the structures as well as how the pattern recognition has been performed for each descriptor. All the structures used in the definition of the structural motifs, regardless of the underlying descriptor used, were obtained from the RCSB PDB database on January 31, 2018. Note that the PDB contains redundant entries, i.e., protein structures with very similar sequences. These redundant structures were included in our analyses, and so the resulting models are biased according to the redundancies of the PDB.

### 2.1. Hydrogen Bond Definitions

As a first benchmark of the application of automatic pattern recognition schemes to (bio)polymers, we consider the case of the hydrogen bond (HB). While there is no shortage of alternative hydrogen-bond definitions based on structure, and PAMM has already been applied to the identification of HBs in water and ammonia (Gasparotto and Ceriotti, 2014; Gasparotto et al., 2016), proteins offer a test case that is more chemically diverse and one for which concrete definitions have been proposed. The latter makes it possible to establish a comparison between our automatic pattern recognition schemes and established categorical descriptions.

#### 2.1.1. Hydrogen Bond Data Selection

The downside of using experimentally determined structures as the basis of our analysis is that the precision of the structural determination—particularly for hydrogen atoms—is limited and varies greatly between entries in the PDB. Given that hydrogen positions are obviously central to the definition of a hydrogen bond motif, only protein crystal structures obtained by X-ray diffraction with a resolution better than 1.2 Å and that included hydrogen atom positions were considered viable. Only 872 structures in the PDB met these requirements and could be properly parsed. Given that each structure contains hundreds of hydrogen bonds, this amount of data was sufficient for our statistical analysis.

From each protein structure, we considered only N, O, and H atoms with occupancy ≥ 0.95. Any oxygen and hydrogen atoms belonging to water or other small molecules were excluded. Four different hydrogen bond flavors were examined, depending on the nature of donor and acceptor: (1) N − H···N; (2) N − H···O; (3) O − H···O; (4) O − H···N.

#### 2.1.2. Geometry Descriptors

For the determination of hydrogen bonding motifs, we examined all triplets of atoms, where one (O or N) is considered as the putative donor, one (O or N) is considered as the putative acceptor, and one is the H atom taking part in the bond. We considered separately the cases in which O and N act as either the donor or the acceptor, i.e., N − H···N, N − H···O, O − H···O, O − H···N. We did not use any additional criterion to identify which atoms could be part of a hydrogen bond, which means that the analysis considers as putative hydrogen bonds also triplets in which the three atoms are chemically bound or adjacent to one another in the backbone or in a side chain. Most of the traditional definitions of hydrogen bonds would implicitly discard these configurations and not consider them altogether. While it would be straightforward to eliminate such configurations as a preliminary step to our analysis, we retained them to serve as a demonstration of the robustness of using PAMM for identifying distinct structural patterns.

Even in protein structures obtained from high-resolution X-ray diffraction, hydrogen positions are often “refined.” In other words, each hydrogen atom is often fixed at a predetermined distance from the atom to which it is covalently bound (Watkin, 2008; Cooper et al., 2010). To ensure that this artificial feature would not further bias the clustering, only the donor–acceptor and acceptor–hydrogen distances were chosen as geometrical descriptors for each hydrogen bond. Ignoring the donor–hydrogen distance does not limit the resolving power of a PAMM analysis, but makes it impossible to automatically eliminate some configurations with a very large donor–hydrogen distance. For this reason, before proceeding with the clustering, we further filtered the hydrogen bonds using the same geometric criteria that has been used in earlier studies of hydrogen bonding in water (Gasparotto and Ceriotti, 2014; Gasparotto et al., 2016), which relies on all of the donor–acceptor, donor–hydrogen, and acceptor–hydrogen distances (*d*_*DA*_, *d*_*DH*_, and *d*_*AH*_, respectively). Those triplets in which the sum of *d*_*DH*_ and *d*_*AH*_ was greater than 4.5 Å were discarded in addition to those in which *d*_*DH*_ was greater than *d*_*AH*_. The latter refinement reduces redundancies when examining different hydrogen bond flavors, as a given triplet with *d*_*DH*_>*d*_*AH*_ in N − H···O is equivalent to that same triplet with *d*_*DH*_ < *d*_*AH*_ in O − H···N; the donor and acceptor labels have just been interchanged. With these conditions, we identified several hundred thousand potential N − H···N and N − H···O triplets and 40–60 thousand O − H···O and O − H···N triplets that we retained for further analysis.

#### 2.1.3. Clustering Parameters

To reduce the computational cost of the procedure while sampling all relevant values of the *d*_*DA*_ and *d*_*AH*_ distances we selected a sparse grid of 2000 configurations on which we computed a kernel density estimation of the probability distribution of different motifs. An approximately uniform distribution of grid points is achieved using a well-established farthest-point sampling (FPS) scheme (Ceriotti et al., 2013). The kernel bandwidth and local scale factors were determined automatically as discussed in Gasparotto et al. (2018). The automatically determined bandwidth was scaled by a factor of 0.3 to account for the strong multi-modality of the distribution, while we found the automatic choice of quick-shift distance to be appropriate. Clusters with weights less than 10^−5^ in the resulting mixture model were discarded, as they were sparsely populated and did not meaningfully contribute to the overall probability distribution and could be considered outliers.

#### 2.1.4. Probabalistic Motif Indentifiers (PMIs)

For each hydrogen bond flavor, the PMI *f*(**x**) at a point **x** = (*d*_*AH*_, *d*_*DA*_) is calculated as in Gasparotto and Ceriotti (2014) and Gasparotto et al. (2018),

(1) $$\begin{array}{r} f\left(\text{x}\right)=\frac{p_{HB}G\left(\text{x}|{\text{μ}}_{HB},{\text{Σ}}_{HB}\right)}{P\left(\text{x}\right)+\zeta }, \end{array}$$

where *p*_*HB*_ is the weight of the Gaussian *G* with mean **μ**_*HB*_ and covariance **Σ**_*HB*_ describing the hydrogen bond, ζ is the background parameter, set to 10^−5^ for our purposes, and *P*(**x**) is the total probability density of the GMM,

(2) $$\begin{array}{r} P\left(\text{x}\right)=\overset{N}{\underset{k}{\sum }}p_{k}G\left(\text{x}|{\text{μ}}_{k},{\text{Σ}}_{k}\right), \end{array}$$

where *N* is the total number of clusters in the model.

The PMI for a distance–angle geometry-based definition of the hydrogen bond is:

(3) $$f(x)=\{\begin{array}{ll} 1, & d_{DA}<3.5\text{ Å},d_{AH}<\text{2}.\text{5Å},\;d_{DH}<\text{1}.\text{5}\\ & \text{Å},∠ADH<\text{30}.{\text{0}}^{{}^{\circ}}\\ 0, & \text{else} \end{array}$$

As another example, the DSSP (Kabsch and Sander, 1983) definition of an N − H···O hydrogen bond, which is based on the distances *d* between the atoms participating in the C = O bond of one residue and the N − H bond of another residue, can also be used to construct a PMI.

To construct the DSSP-based PMI, we computed the required DSSP distances for all {N, H, C, O} quadruplets in each protein for which all four atoms have occupancy ≥ 0.95, and map the quadruplet to (*d*_*AH*_, *d*_*DA*_) space simply by taking *d*_*AH*_ as the oxygen–hydrogen distance and *d*_*DA*_ as the nitrogen–oxygen distance. Then for each **x** = (*d*_*AH*_, *d*_*DA*_), we computed the joint probability distribution

(4) $$\begin{array}{r} P_{HB}\left(\text{x}\right)=P\left(\text{x},E_{DSSP}<-0.5\;\text{kcal/mol}\right), \end{array}$$

where *E*_*DSSP*_ is the DSSP electrostatic energy as defined in Kabsch and Sander (1983). The DSSP-based PMI can then be constructed following Equations 1, 2 by replacing *G*(**x**|**μ**_*HB*_, **Σ**_*HB*_) with the joint probability density *P*_*HB*_(**x**) and by defining the total probability density as

(5) $$\begin{array}{r} P\left(\text{x}\right)=p_{HB}P_{HB}\left(\text{x}\right)+\left(1-p_{HB}\right)P\left(\text{x},E\geq -0.5\;\text{kcal/mol}\right). \end{array}$$

where the weight *p*_*HB*_ is the fraction of C = O, N − H pairs that have *E* < −0.5 kcal/mol. It should be noted that the DSSP PMI is based on only a subset of the data used to define the PAMM PMIs and contains approximately 550,000 N − H···O triplets. As stated in 2.1.1 in the Methods, we discarded atoms from the analysis that had an occupation less than 0.95 in order to train PAMM on unambiguous atomic geometries. This, combined with the fact that the DSSP definition requires the positions of C atoms, means that the DSSP PMI was built considering only (C = O, N − H) pairs in the protein backbone for which each of the C, O, N, and H atoms had an occupation < 0.95, narrowing the dataset.

In order to compare different HB definitions and to quantify how often they disagree in identifying a local motif in **x** = (*d*_*AH*_, *d*_*DA*_) space as an HB, we introduce the quantity

(6) $$\begin{array}{r} \delta_{AB}=\frac{1}{\lambda }\frac{\int P_{total}\left(\text{x}\right)f_{A}\left(\text{x}\right)f_{B}\left(\text{x}\right)d\text{x}}{\int P_{total}\left(\text{x}\right)\left[f_{A}\left(\text{x}\right)+f_{B}\left(\text{x}\right)-f_{A}\left(\text{x}\right)f_{B}\left(\text{x}\right)\right]d\text{x}}, \end{array}$$

which is the probability that the PMIs *A* and *B* both identify point **x** as an HB relative to the probability that either one or the other identify an HB. *P*_*total*_(**x**) is the total probability distribution of observing (*d*_*AH*_, *d*_*DA*_) in the PDB dataset across all hydrogen bond flavors. The normalization factor λ is included to account for the fact that the PMIs *f* are posterior probabilities rather than true probability distributions. Thus, λ is chosen such that Equation 6 is equal to one when *f*_*A*_(**x**) = *f*_*B*_(**x**):

(7) $$\begin{array}{r} \text{λ}=\sqrt{\frac{\int P_{total}\left(\text{x}\right)f_{A}^{2}\left(\text{x}\right)d\text{x}}{\int P_{total}\left(\text{x}\right)\left[2f_{A}\left(\text{x}\right)-f_{A}^{2}\left(\text{x}\right)\right]d\text{x}}\cdot \frac{\int P_{total}\left(\text{x}\right)f_{B}^{2}\left(\text{x}\right)d\text{x}}{\int P_{total}\left(\text{x}\right)\left[2f_{B}\left(\text{x}\right)-f_{B}^{2}\left(\text{x}\right)\right]d\text{x}}} \end{array}$$

### 2.2. Dihedral Angles for Secondary Structure Recognition

Secondary-structure patterns play a central role in rationalizing the structure and behavior of proteins. Well-established definitions exist based on the identification of HBs along the protein backbone, such as STRIDE (Frishman and Argos, 1995) and DSSP (Kabsch and Sander, 1983). There is, however, a need for definitions of secondary structure that are based on continuous structural coordinates, for instance, to bias atomistic simulations or to perform structure searches (Pietropaolo et al., 2008; Pietrucci and Laio, 2009). As an example of how one can use PAMM to provide a definition of secondary structure motifs that is based on a simple, local representation of the backbone, we used the Ramachandran dihedrals (Ramachandran et al., 1963), whose strong correlation to secondary structure has been long appreciated (Hollingsworth et al., 2012; Wood and Hirst, 2005; Kountouris and Hirst, 2009).

#### 2.2.1. Dihedral Angle Data Selection

Because the calculation of dihedral angles is not sensitive to hydrogen atomic positions, the PAMM analysis of dihedral angles included all experimental protein crystal structures from the RCSB PDB (as of January 31, 2018) obtained from X-ray diffraction with a resolution better than 1.5 Å, totaling 12,708 structures and 4,275,677 residues from which dihedral angles could be extracted. Note again that no measures were taken to discard redundant structures from the PAMM analysis, hence the resulting mixture model is biased according to the redundancies of the PDB.

#### 2.2.2. Clustering and Secondary Structure Classification

Using PAMM, a GMM of the backbone dihedral angles (ϕ and ψ) calculated with BioPython (Cock et al., 2009) was constructed. We performed a kernel density estimation on 4000 FPS grid points. In this case, we used a bandwidth scaling factor of 0.15, and a scaling of the quick-shift threshold of 0.20, compared to the values determined automatically based on the heuristics discussed in Gasparotto et al. (2018). We found that the automatic parameters were smoothing excessively the distribution, resulting in a loss of resolving power. We determined the optimal parameters by monitoring the number of clusters and their robustness as assessed by a bootstrapping analysis. We also constructed PAMM GMMs based on higher dimensional feature spaces based on chains of ϕ and ψ angles in consecutive residues. Here we again used 4000 grid points but selected a bandwidth scaling factor of 0.30 and set the quick-shift scaling to 0.80. Similar to the case of the HB, we discarded clusters with weights < 10^−5^.

#### 2.2.3. Comparison of Secondary-Structure Definitions

Given that each point **x** = (ϕ, ψ) corresponding to a single amino acid residue is associated with a secondary structure classification *y* from DSSP/STRIDE and a cluster assignment *A* with probability *p*^(*A*)^(**x**) from PAMM, a joint probability distribution *P*(*A, y*) can be constructed by summing the cluster probabilities over all points **x**_*y*_ with secondary structure *y*,

(8) $$\begin{array}{r} P\left(A,y\right)=\frac{1}{N}\underset{{\text{x}}_{y}}{\sum }p^{\left(A\right)}\left({\text{x}}_{y}\right), \end{array}$$

where *N* is the total number of residues considered. *P*(*A, y*) characterizes completely the relationship between the two definitions. Based on the joint probability we can compute the marginals *P*(*A*) and *P*(*y*) and the conditional probabilities *P*(*A*∣*y*) and *P*(*y*∣*A*), which provide equivalent information and make it easy to identify the correspondence—if any—between the PAMM-based PMI and the conventional definitions. For reference, the DSSP and STRIDE secondary structure classifications are as follows: B, isolated β-bridge; E, extended strand; G, 3_10_-helix; H, α-helix; I, π-helix; T, turn; S, bend (DSSP only); C, loop, irregular element, or none of the above (“coil”). We use an “X” to signify an amino acid residue for which no secondary structure was assigned.

One can summarize the ability of the automatic definition to reproduce the classification given by STRIDE or DSSP by viewing the joint probability *P*(*A, y*) in the framework of the Q3 (or Q8) accuracy score (Rost and Sander, 1993a). Given a particular clustering arrangement, one or more clusters can be selected that individually correspond to strands (B, E), helices (G, H, I) or coils (C, S, T) by assigning each cluster *A* the secondary structure that maximizes *P*(*y*∣*A*).

Thus, for sets of clusters E,H,C corresponding to strands, helices, and coils, the Q3 score is the sum $Q_{E}+Q_{H}+Q_{C}$, where

(9a) $$\begin{array}{r} Q_{E}=\underset{i\in E}{\sum }\left(P\left(i,B\right)+P\left(i,E\right)\right) \end{array}$$

(9b) $$\begin{array}{r} Q_{H}=\underset{j\in H}{\sum }\left(P\left(j,G\right)+P\left(j,H\right)+P\left(j,I\right)\right) \end{array}$$

(9c) $$\begin{array}{r} Q_{C}=\underset{k\in C}{\sum }\left(P\left(k,C\right)+P\left(k,S\right)+P\left(k,T\right)\right), \end{array}$$

and the secondary structure assignments B, E, G, H, I, C, S, and T are those determined by DSSP or STRIDE.

### 2.3. Smooth Overlap of Atomic Positions Representation

The analysis protocols that we have discussed above identify the presence of significant motifs based exclusively on how often a given local atomistic environment occurs in a reference dataset. While this procedure makes it possible to rely on simple and rather generic descriptors of local structure, it still requires a dose of chemical intuition, i.e., it is necessary to know the basis of hydrogen bonding and that dihedral angles can be used to identify the secondary structure of a protein. To fulfill our goal of creating a completely agnostic framework, one would need to use a more abstract, generally applicable measure of the atomistic environment that does not require any chemical intuition. To this end, we have employed SOAP, a method that can represent each chemical environment in a complete way and that can be applied seamlessly to any system, from biomolecules to materials.

#### 2.3.1. Brief Introduction to SOAP

Before explaining the clustering procedure and parameters used with SOAP, we briefly introduce the representation. This is not meant to be a complete introduction, and we redirect the interested reader to more detailed papers previously published on the topic (Bartók et al., 2013; Bartók and Csányi, 2015; De et al., 2016). The SOAP vector is a recently introduced, atom-centered, density-based representation that has been used in many applications, from solids to molecular systems. It has been proven useful in describing and predicting many atomic and molecular properties such as structure and energy (De et al., 2016). The SOAP framework represents the atomic density around an atom *j* as a sum of Gaussians centered on each surrounding atom of species α. The sum can be cast into a smooth, local probability amplitude $\psi_{X_{j}}^{\alpha }\left(\text{r}\right)$ by employing a cutoff function *f*_*c*_ that determines the extent of the local environment:

(10) $$\begin{array}{r} \text{〈}\alpha \text{r}|X_{j}\text{〉}\equiv \psi_{X_{j}}^{\alpha }\left(\text{r}\right)=\underset{i\in \alpha }{\sum }f_{c}\left({\text{r}}_{ij}\right)g\left(\text{r}-{\text{r}}_{ij}\right). \end{array}$$

The main parameters determining the behavior of the SOAP features are the cutoff distance—which defines the range of structural correlations that are deemed to be relevant—and the width of the Gaussian functions—which determines the sensitivity to atomic displacements.

In the original formulation of SOAP (Bartók et al., 2013), the atom density is expressed by expanding the environmental density in a basis of orthogonal radial basis functions *R*_*n*_(*r*) and spherical harmonics $Y_{m}^{l}\left(\hat{\text{r}}\right)$,

(11) $$\begin{array}{r} \text{〈}\alpha nlm|X_{j}\text{〉}=\int \text{d}\text{r}R_{n}\left(r\right)Y_{m}^{l}\left(\hat{\text{r}}\right)\text{〈}\alpha \text{r}|X_{j}\text{〉}. \end{array}$$

This amplitude is invariant to translations in addition to permutations of atoms within each species α, but it is not invariant to rotations. Rotation invariance can be achieved by integrating the overlap between two atomic environments X over all relative rotations $\hat{R}$, yielding the kernel,

(12) $$\begin{array}{r} K^{\left(\nu \right)}\left(X_{j},X_{k}\right)=\int \text{d}\hat{R}{\text{〈}X}_{j}|R|{X_{k}\text{〉}}^{\nu }. \end{array}$$

For ν = 2, the kernel is equivalent to the scalar product between the power spectra of environments *j* and *k*,

(13) $$\begin{array}{r} K^{\left(2\right)}\left(X_{j},X_{k}\right)=\underset{\alpha n\alpha^{\prime }n^{\prime }l}{\sum }{\text{〈}X}_{j}|\alpha n\alpha^{\prime }n^{\prime }l\text{〉}\text{〈}\alpha n\alpha^{\prime }n^{\prime }l|X_{k}\text{〉}. \end{array}$$

The power spectrum vectors $\alpha n\alpha^{\prime }n^{\prime }l|X_{k}$ can be used as an explicit, general, and complete representation of chemical environments.

#### 2.3.2. SOAP Data Selection

Although SOAP is a powerful descriptor, the high dimensionality of the SOAP vectors $\alpha n\alpha^{\prime }n^{\prime }l|X_{k}$ makes PAMM pattern recognition based on these descriptors computationally intractable for large datasets. Therefore, we first performed a Principal Component Analysis (PCA) of the SOAP vectors with the aim of reducing the dimension of the input space for PAMM while maintaining the most discriminating SOAP features of the individual proteins. To accelerate the process, we used an FPS subset of SOAP components to reduce the input space for the PCA while maintaining its span. In particular, we selected 100 random structures from the same set used in the dihedral angle clustering and computed the SOAP vectors for all of the C_α_ atoms in the selected structures, taking into consideration all C, N, and O atoms within a cutoff radius of 6.0 Å as part of the local environment, which is large enough to incorporate information on several neighboring residues. From this collection of SOAP vectors, we selected 200 SOAP components via FPS, using the squared Euclidean distance between the SOAP vectors as the measure of separation (Imbalzano et al., 2018).

The SOAP vectors centered around all C_α_ atoms were then computed for all structures just as they were for the random subset, but only the FPS-selected components were kept and used to build the PCA representation; all other components of the SOAP vector were discarded. The full parameters used to generate the SOAP vectors are given in the Supplemental Material.

#### 2.3.3. Clustering and Classification

The first 2, 6 and 10 PCA components of the reduced SOAP vectors were clustered by PAMM using 4000 grid points and a quick shift parameter of 1.0. The Kernel Density Estimation bandwidth scaling factor was chosen to be 0.20, 0.50, and 0.80 for the 2, 6, and 10 PCA component representations respectively. Clusters with weights < 10^−5^ were discarded.

#### 2.3.4. Probability Distribution

Because each individual reduced SOAP vector is based on an expansion around the C_α_ atoms, each vector corresponds to a single residue and therefore can be associated with a DSSP- or STRIDE-assigned secondary structure. The joint and conditional probability distributions for the reduced SOAP vectors clustered by PAMM were computed in the same manner as those for the dihedral angles, as were the Q3 and Q8 scores relative to DSSP and STRIDE.

### 2.4. Supervised Classification

Given that the SOAP representation can be tuned to encompass environments of different sizes and provide a complete description of the correlation between atomic positions, it gives us an opportunity to verify whether any discrepancy between the PAMM classification and the reference heuristics is due to the fact that the truncated representations that we use are incomplete, or due to the fact that the reference heuristics are not reflected in the probability distribution of motifs in the PDB. We can assess the completeness of the representations by training a *supervised* model to recognize DSSP or STRIDE motifs; that is, we can associate the SOAP description of the atomic environment $X_{i}$ of each C_α_ atom with the label *y*_*i*_ assigned to it by DSSP or STRIDE. To perform this classification task we used a support vector machine (SVM) (Cortes and Vapnik, 1995) as implemented in the scikit-learn Python package (Pedregosa et al., 2011) to perform multiclass classification of a PCA of SOAP environments $X_{i}$ according the labels *y*_*i*_. For comparison, SVMs using backbone dihedral angles were also constructed. The SVMs employed a “one vs. one” classification scheme (Knerr et al., 1990) with a Gaussian kernel with width γ = 1/*N*_*f*_, where *N*_*f*_ is the number of features, and regularization parameter *C* = 1.0. Furthermore, the SOAP PCA and dihedral angle data were scaled to have zero mean and unit variance before building the SVM. Of the approximately 4.3 million residues present in our dataset, we selected 200,000 residues at random (excluding those that were not assigned a secondary structure by DSSP or STRIDE) to train and evaluate the SVM. Of these 200,000 residues, 50,000 were randomly selected to serve as the training set, and the remaining 150,000 served as the test set. The asymptotic (large train set size) classification accuracy of the supervised model indicates the limit that can be achieved with a given environment representation. Learning curves of the Q3 and Q8 scores for the SVM are provided in the Supplemental Material.

## 3. Results and Discussion

### 3.1. Hydrogen Bonds

**Figure 1:**
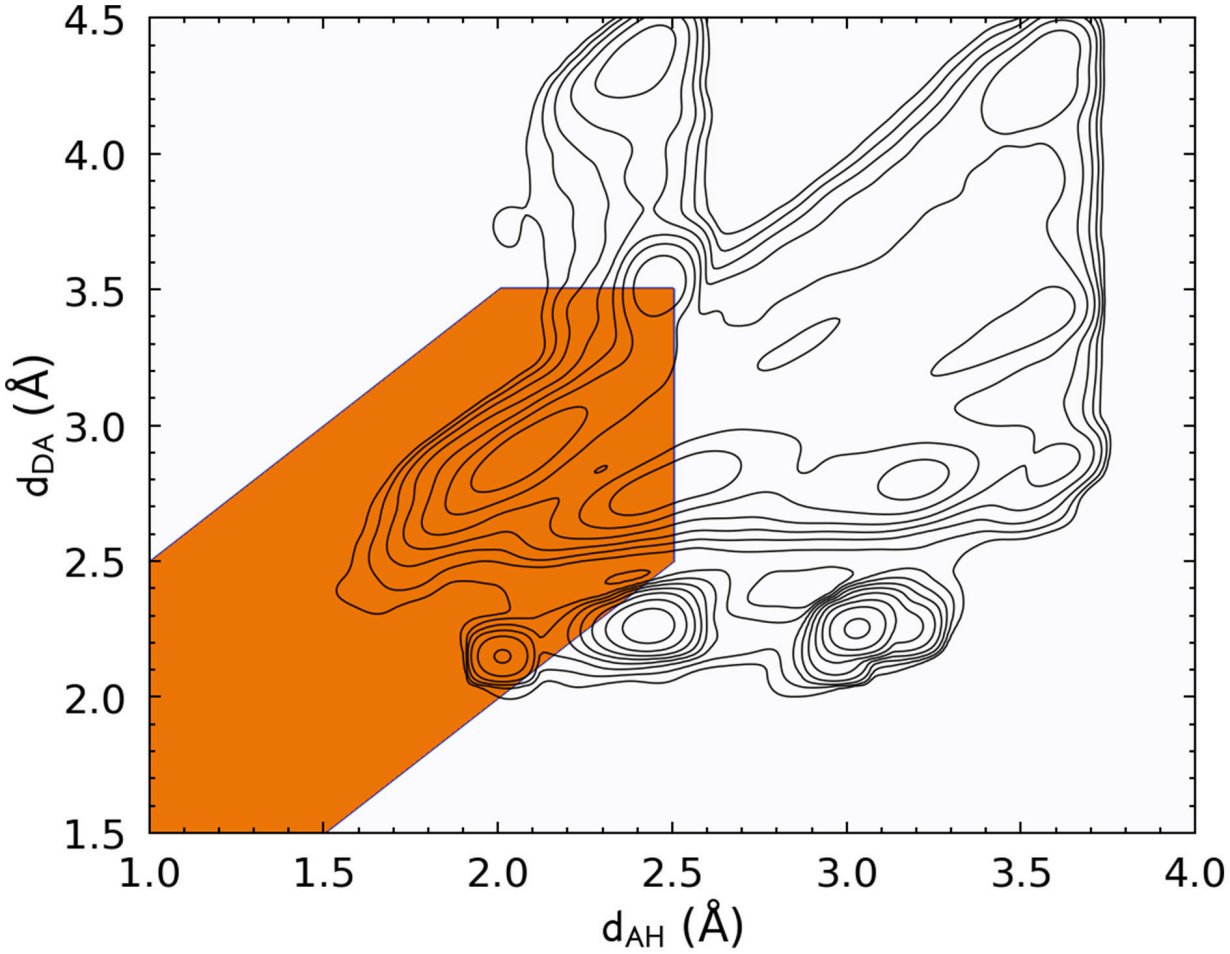
Histogram of the acceptor–hydrogen and donor–acceptor distances across all hydrogen bond flavors, plotted with log-spaced contours. The maximum at (*d*_*AH*_ ≈ 2.1Å, *d*_*DA*_ ≈ 2.8Å) corresponds to the typical H-bond range. Other maxima are associated with other structural features, such as covalently bound groups on the side chains, geometries in which the two electronegative atoms are in the same residue, or configurations in which the hydrogen atom is not bound to the donor. The orange-shaded area corresponds to the distance-angle PMI as defined in Equation 3.

Let us start by discussing the definition of HBs based on a traditional distance–angle criterion. Figure 1 shows the probability distribution of (*d*_*AH*_, *d*_*DA*_) computed by accumulating simultaneously all four kinds of HBs. The PMI associated with the conventional definition of the hydrogen bond is highlighted. This definition encompasses a large peak in *P*(**x**) that indeed corresponds to hydrogen-bonded configurations, but it also includes several additional peaks. By inspection, we found that these additional modes of the distribution are associated with motifs in which the putative donor and acceptor atoms are part of the same amino acid residue or where the H atom is not chemically bound to the donor. In practice, these geometries would be discarded a priori because most codes for analyzing biomolecular data take covalent bonding information into account. Figure 1, however, underscores the complex heuristics that are necessary to apply well-established definitions of atomic-scale motifs, and serves as a warning of the risks one could incur when blindly following these prescriptions in a different context than the usual forcefield simulations in which the chemical connectivity is fixed.

**Figure 2:**
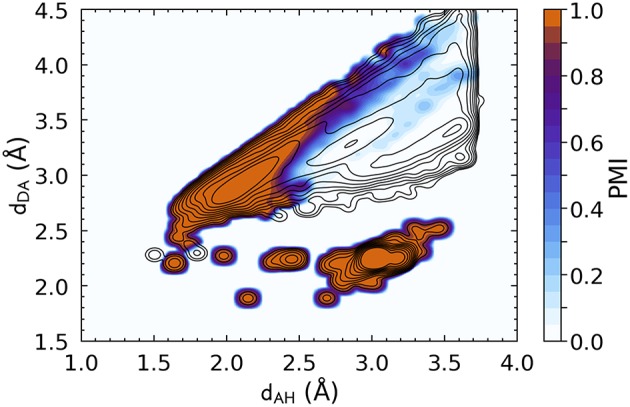
Density plot of the PMI constructed using the DSSP hydrogen bond definition with ζ = 10^−5^. The PMI is plotted on top of a histogram of the distance features for N − H···O hydrogen bonds (discarding non-backbone groups, and any triplet for which it is not possible to define a DSSP H-bond energy, e.g., due to partial occupations), with log-spaced contours. DSSP identifies very clearly the H-bond peak, but also picks up spurious correlations corresponding to immediately adjacent residues [peak at (*d*_*AH*_ ≈ 3.0, *d*_*DA*_ ≈ 2.25)].

Similar considerations apply to the DSSP definition, whose corresponding PMI is shown in Figure 2. The DSSP definition follows more closely the main HB peak of the distribution, as one would expect given that it is heavily fine-tuned for one specific flavor of bond, N − H···O, between peptide groups. At the same time, DSSP also requires further heuristics to discard spurious correlations corresponding to N − H and C = O in immediately adjacent residues, where (*d*_*AH*_ ≈ 3.0, *d*_*DA*_ ≈ 2.25).

**Figure 3:**
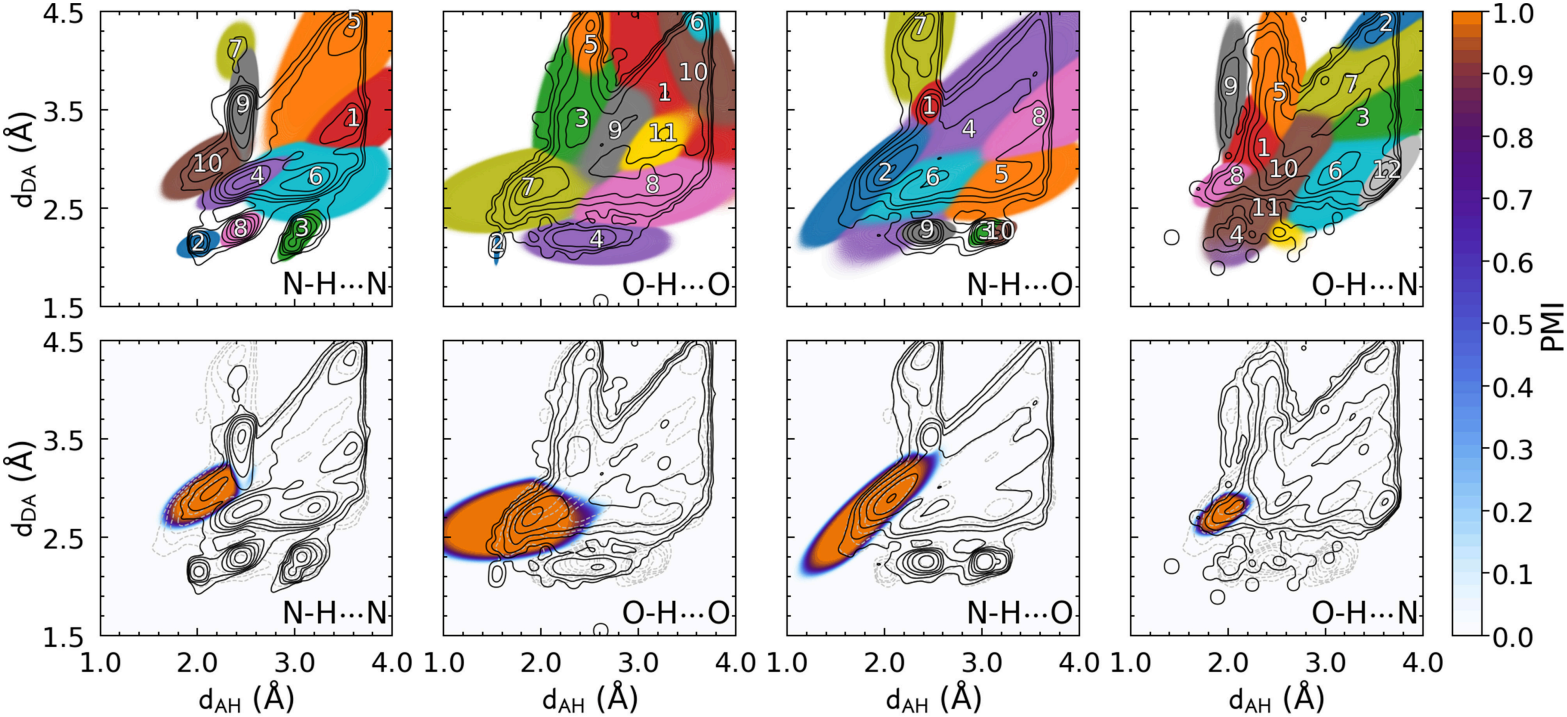
The top panels represent all the clusters identified by PAMM. Clusters are numbered in an arbitrary order, and the colors reflect the cluster that is dominant in each region, as determined by its corresponding PMI [as defined in Equation (1), computed with ζ = 10^−5^]. Bottom panels highlights the PMI of the cluster associated with the hydrogen bond.

Contrast these figures with the top row of Figure 3, which shows the PAMM PMIs for each cluster in the GMMs, computed separately for the four hydrogen bond flavors. The four distributions differ substantially from each other, and from the overall *P*(**x**), while exhibiting multiple modes that are correctly identified by PAMM and assigned different cluster indices. Some of these modes correspond to correlations between covalently bound atoms, while others correspond to longer-range intermolecular correlations. For each flavor, the cluster that corresponds to the hydrogen bond is that with its center (mode) nearest to (*d*_*AH*_ = 1.82 Å, *d*_*DA*_ = 2.74 Å) (Gasparotto and Ceriotti, 2014). The corresponding PMIs, which are plotted in the bottom row of Figure 3, identify with great precision the region in the probability distribution that corresponds to the HB, and eliminate automatically the spurious configurations due to adjacent residues or covalently bound groups without the need for additional heuristics.

Figure 3 also shows that different kinds of hydrogen bonds correspond to noticeably different portions of (*d*_*AH*_, *d*_*DA*_) pattern space (a figure comparing different definitions is shown in the Supplemental Material). This means that a substantial fraction of molecular patterns would be misclassified if one tried to transfer the definition between different kinds of HB. As shown in Table 1, the probability that two definitions yield the same classification, as measured by Equation 6, can be as low at 50%. The agreement between the data-driven PMIs and the conventional distance–angle definition is even poorer, as shown in Table 2 and in Figure S2 in the Supplemental Material. It should be stressed, however, that this is largely due to the inclusion of correlations that are usually discarded by additional heuristics: if one computes the PMI similarity using a probability distribution *P*_*total*_(**x**) that discards atoms in the same or nearby residues, the probability increases substantially, particularly for N − H···N and N − H···O, as these are the flavors are responsible for the majority of spurious hydrogen bond geometries (e.g., intra-arginine or intra-histidine N − H···N triplets and backbone N − H···O triplets with donor and acceptor atoms in directly adjacent residues). The increase in PMI similarity is generally less pronounced when comparing two different hydrogen bond flavors because these PMIs are derived from a PAMM GMM, which automatically recognizes the spurious geometries as separate motifs. This example, although simple, demonstrates how one can use data-analytic techniques to extract definitions of molecular motifs based on experimental structural data. It also serves as a reminder of how heuristic definitions can lack transferability, and how their apparent simplicity is often contingent on a considerable amount of prior knowledge and the enforcement of additional conditions.

**Table 1:** Probabilities that two PMIs corresponding to different hydrogen bond flavors agree that a point is a hydrogen bond (Equation 6).

**PMI A**	**PMI B**	**δ_*AB*_**	**$\delta_{AB}^{\left(i\right)}$**	**$\delta_{AB}^{\left(i+1\right)}$**
N − H···N	N − H···O	0.92	0.93	0.94
N − H···N	O − H···O	0.57	0.63	0.74
N − H···N	O − H···N	0.60	0.59	0.60
O − H···O	N − H···O	0.55	0.61	0.71
O − H···O	O − H···N	0.60	0.68	0.85
N − H···O	O − H···N	0.57	0.57	0.58

*The superscripts (i) and (i+1) correspond to probabilities δ_AB_ where P_total_(**x**) excludes donor–hydrogen–acceptor triplets in which the donor and acceptor atoms are in the same residue (i), or additionally in adjacent residues (i+1)*.

**Table 2:** Probabilities that the hydrogen bond PMI and the distance–angle definition agree that a point is a hydrogen bond (Equation 6).

**Bond type**	**δ_*AB*_**	**$\delta_{AB}^{\left(i\right)}$**	**$\delta_{AB}^{\left(i+1\right)}$**
N − H···N	0.56	0.65	0.89
N − H···O	0.60	0.71	0.93
O − H···O	0.63	0.65	0.68
O − H···N	0.33	0.39	0.53

*The superscripts (i) and (i+1) correspond to probabilities δ_AB_ where P_total_(**x**) excludes donor–hydrogen–acceptor triplets in which the donor and acceptor atoms are in the same residue (i), or additionally in directly adjacent residues (i+1)*.

### 3.2. Dihedral Angles and Protein Secondary Structure

As another example of using simple geometric descriptors to find and evaluate atomic-scale motifs, we used PAMM to automatically detect dihedral angle motifs in proteins. Backbone dihedrals are central to our understanding of protein structure (consider, for example, the widespread use of the Ramachandran plot), and provide a rather unbiased description of a polymer chain that could be easily applied to other classes of polymers, whose structure is determined by different kinds of interactions.

**Figure 4:**
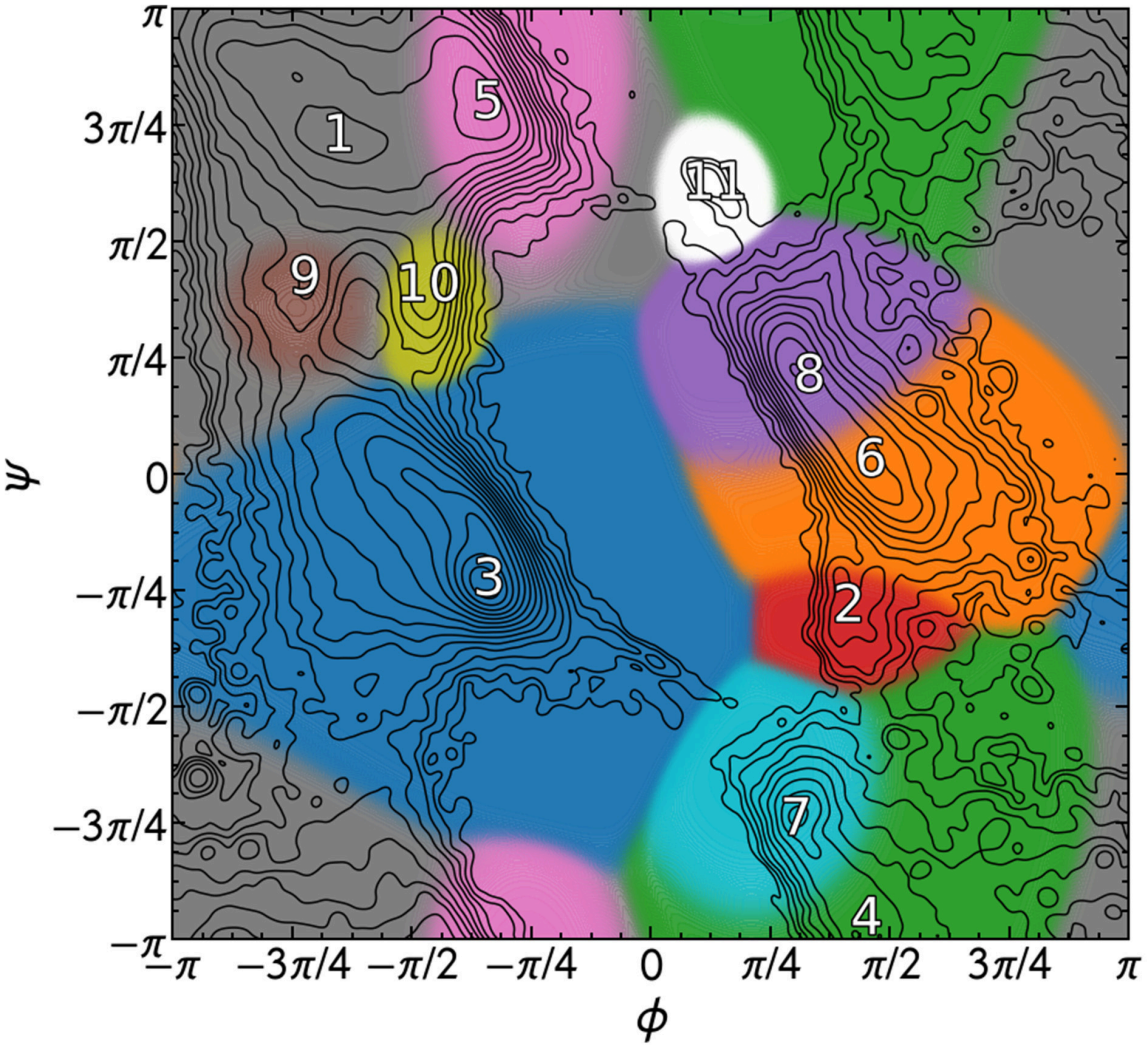
PAMM clustering of all calculated dihedral angles with ζ = 0. Cluster numbers are placed at the mode of the cluster, and each cluster has been colored differently. The isocontours of the total distribution are equally spaced on a logarithmic scale.

**Figure 5:**
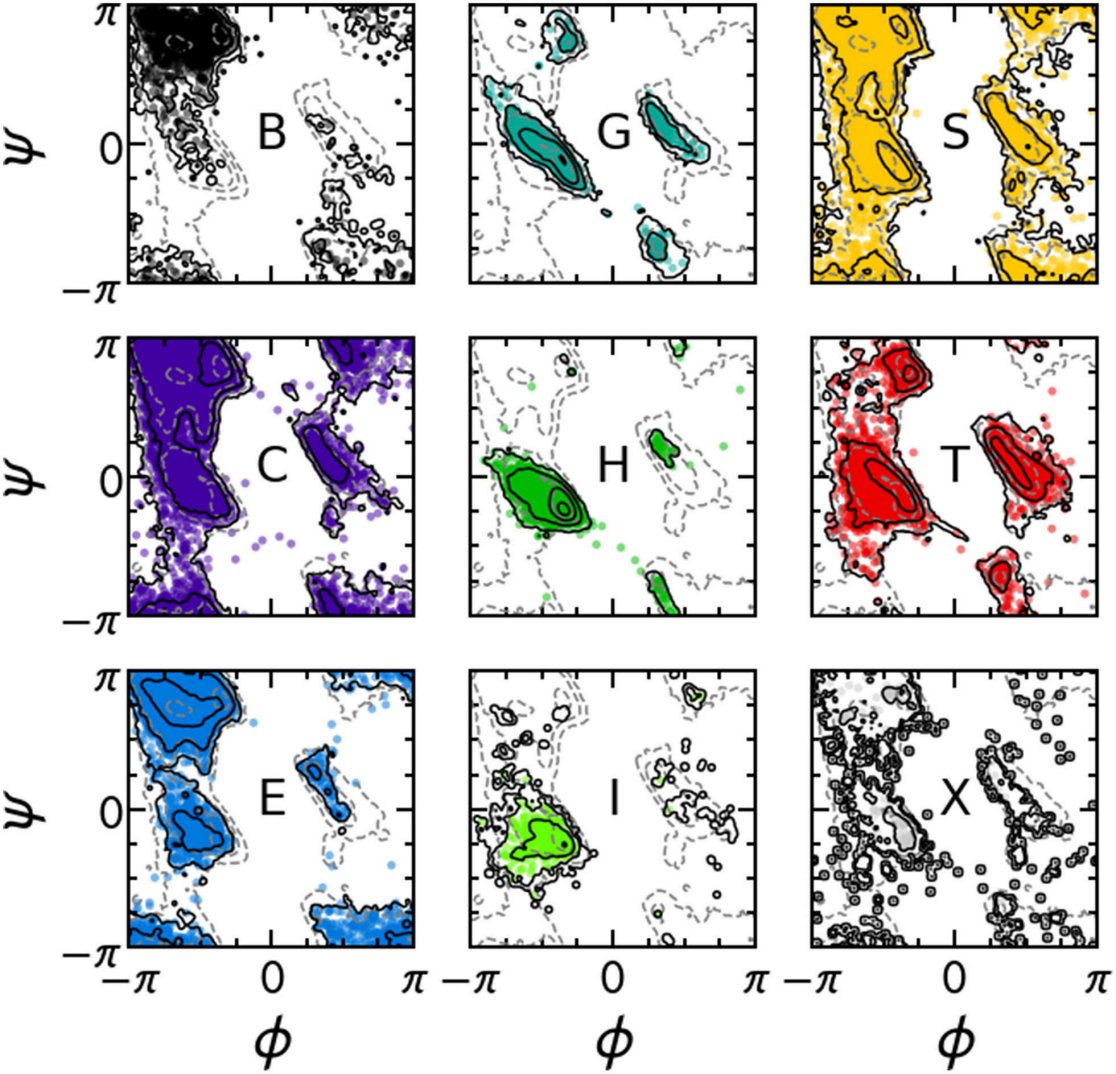
Collection of 100,000 randomly selected (ϕ, ψ) pairs, separated according to the DSSP secondary structure classification of each pair. Solid contours correspond to the distribution of the secondary structure of interest; dashed contours correspond to the total distribution of all ϕ, ψ angles. Contours are equally spaced on a logarithmic scale.

The PMIs for each of the Gaussians in a PAMM GMM of the dihedral angles ϕ and ψ are shown in Figure 4. The PAMM dihedral angle clustering agrees well with those obtained by Hollingsworth et al. (2012) and Nagy and Oostenbrink (Nagy and Oostenbrink, 2014), who have previously developed classification schemes based solely on dihedral angles. However, we observe like Hollingsworth et al. that dihedral angle patterns do not necessarily correspond to established secondary structure definitions, which is made clear upon comparison of Figure 5, which shows 100,000 randomly selected dihedral angle pairs colored according to their DSSP and STRIDE secondary structure assignments, and the clusters presented in Figure 4. As we will discuss further down, failure of dihedral angles to match established secondary-structure classifications is not due to an intrinsic lack of resolving power, but to the fact that dihedrals emphasize different kinds of structural correlations, so that secondary structure motifs are not associated with separate modes in feature space.

**Figure 6:**
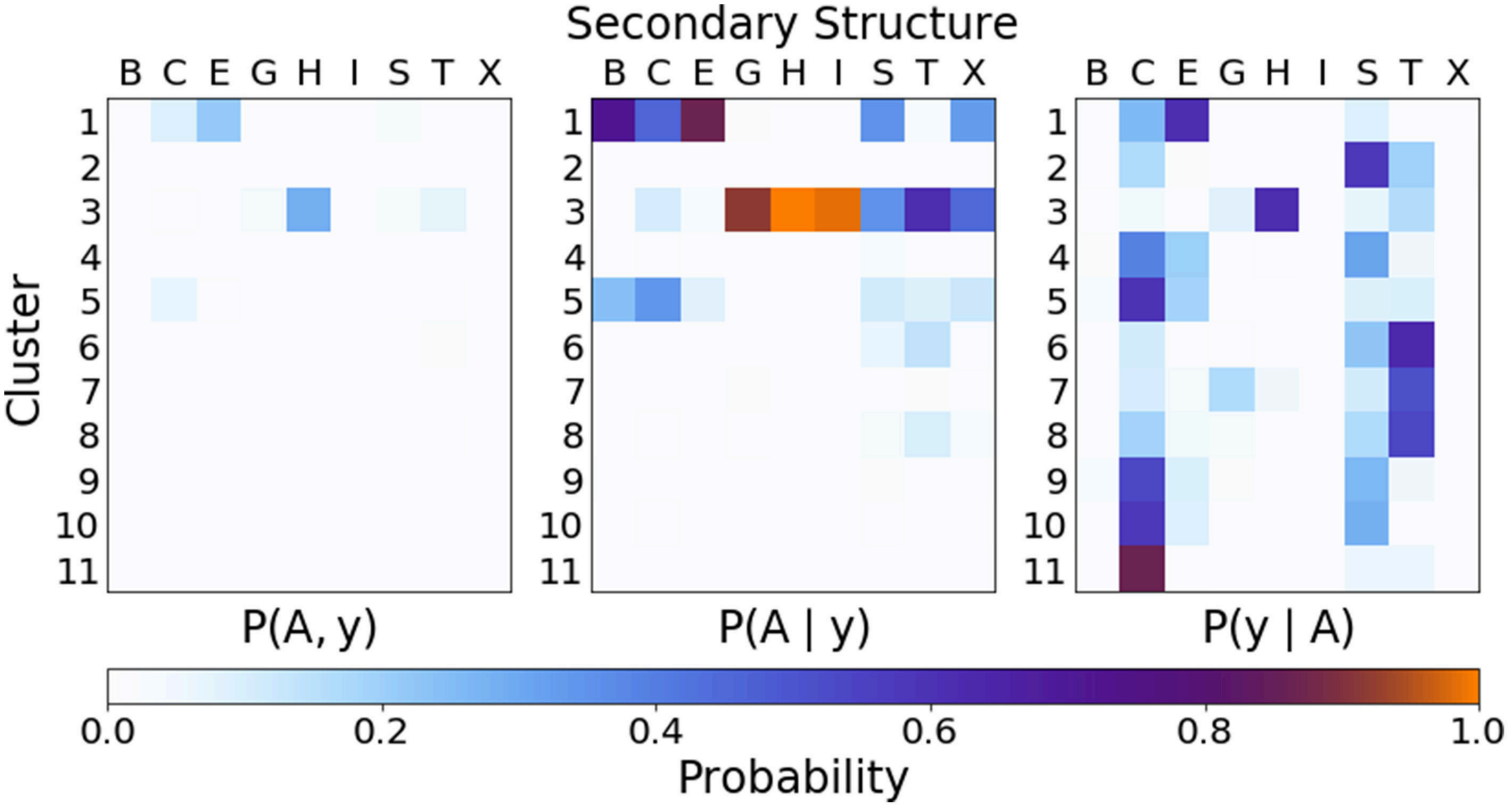
Joint and conditional probabilities for the secondary structures obtained from DSSP and the clustering of dihedral angles from PAMM, where *A* is the cluster assignment and *y* the secondary structure classification.

In order to quantify the correspondence between the PAMM cluster assignment and the secondary structure assignment, the joint and conditional probability distributions as outlined in section 2.2.3 were computed. Figure 6 gives the joint and conditional probability distributions of the PAMM cluster assignment and the DSSP secondary structure assignment. (The probability distributions using the STRIDE secondary structure assignment are very similar to those using the DSSP assignment, and can be found in the Supplemental Material.)

Figure 6 Shows that there is a strong correlation between the most populated PAMM clusters (labeled by *A*∈{1, …, 11}) and DSSP motifs (labeled by *y*∈{*B, C, E, G, H, I, S, T, X*}), with *A* = 1, *y* = *E* and *A* = 3, *y* = *H* being by large the most probable mutual assignments. The joint probability distribution, however, is not easy to interpret because of the widely varying populations of the different clusters. For this reason, the figure also shows the conditional probabilities, which normalize the joint assignments based on the DSSP [*P*(*A*∣*y*)] and PAMM [*P*(*y*∣*A*)] marginals. This analysis shows that the PAMM Cluster 1 encompasses most of the strand-like motifs (B, E) and Cluster 3 encompasses most of the helices (G, H, I). The distribution conditional on DSSP assignments is also insightful, showing that a large fraction of E and H motifs are assigned to PAMM Clusters 1 and 3, while the distribution conditional on PAMM cluster shows that disordered motifs are more evenly spread across all of the clusters. This comparison suggests that conventional heuristics are consistent with the actual distribution of structures in well-characterized proteins when it comes to well-defined sheet and helical motifs. On the other hand—at least when seen through the lens of the Ramachandran angles—DSSP bends, turns and coils are not clearly identifiable with separate peaks in the observed probability distribution. There are nevertheless clusters that are associated with clear peaks, and that are not associated with helices or strands. This suggests that “disordered” sections of proteins exhibit substantial order on the scale of the conformation of individual residues, and that looking at the statistics and correlations of these local motifs might be a better approach to characterize disordered polypeptides than trying to fit them within the existing categories.

One can further contextualize the probability distributions with the framework of the Q3 or Q8 score. Assigning Cluster 1 (see Figure 4) to the “strand” classification, Cluster 3 to the “helix” classification, and associating all other clusters with the “coil” designation yields a Q3 score of 0.70 relative to DSSP and 0.72 relative to STRIDE.

The rather low value of the Q3 score is comparable to the reported match scores of DISICL (Nagy and Oostenbrink, 2014) (with our PAMM PMI-based method performing better relative to DSSP but more poorly relative to STRIDE), which is also based solely on backbone dihedral angles. However, the Q3 score of our cluster-based secondary structure assignments is substantially lower than other methods that rely on dihedral angles in addition to amino acid sequences (Wood and Hirst, 2005; Kountouris and Hirst, 2009), or C_α_ distances (Martin et al., 2005). In this context, the underperformance of our method in classifying secondary structure could be given two different justifications. One is that the traditional secondary structure motifs are based on rather arbitrary thresholds, that recognize configurations as separate modes even when there are no clearly distinct maxima in the distribution of atomic configurations, regardless of the (reasonable) choice of input representation. Another is that our specific choice of representation, i.e., pairs of backbone dihedrals, is insufficient to distinguish between different motifs because of its excessive locality. The latter hypothesis is supported by the large overlap of different DSSP motifs in dihedral space (Figure 5), and can be tested by using different representations of the atomic motifs as the input to a PAMM analysis.

As a means of including more non-local information into the model while relying on a representation based purely on dihedrals, we also performed a PAMM clustering on the dihedral angles of consecutive residues, comparing the cluster assignment to the DSSP and STRIDE secondary structure classifications of the middle residue in the sequence. Just as in the two-dimensional case, in six dimensions (three consecutive residues) and ten dimensions (five consecutive residues) the helices and strands are localized to one or two clusters, while the other secondary structures are distributed across several clusters (The probability distributions for the six- and ten-dimensional clusterings are given in the Supplemental Material). As a consequence, the Q3 score is largely the same among the two-, six-, and ten-dimensional representations (see Table 3). Moreover, we observe that the Q3 score can be sensitive to the choice of clustering parameters; relatively small changes to the parameters can change the resulting GMM such that the Q3 score increases or decreases by ≈ 0.05–0.10. For example, reducing the quick shift parameter from 0.90 to 0.80 in the ten-dimensional case roughly doubles the number of clusters and the Q3 score increases from approximately 0.68 to 0.73 for both DSSP and STRIDE.

**Table 3:** Q3 and Q8 scores relative to DSSP for PAMM PMI and SVM predictions of secondary structure based on a PCA of SOAP vectors and dihedral angles at various dimensionalities.

	**PAMM PMI**		**SVM**	
**Representation**	**Q3**	**Q8**	**Q3**	**Q8**
ϕ, ψ (2D)	0.71	0.61	0.78	0.67
ϕ, ψ (6D)	0.74	0.63	0.87	0.80
ϕ, ψ (10D)	0.73	0.61	0.88	0.82
SOAP PCA (2D)	0.73	0.58	0.75	0.61
SOAP PCA (6D)	0.72	0.58	0.84	0.73
SOAP PCA (10D)	0.71	0.55	0.90	0.79
SOAP PCA (100D)	—	—	0.95	0.89

*The reported SVM scores are an average over five separate constructions of the SVM, each time using a new random subset of 200,000 residues, with 50,000 of these serving as the training set*.

**Figure 7:**
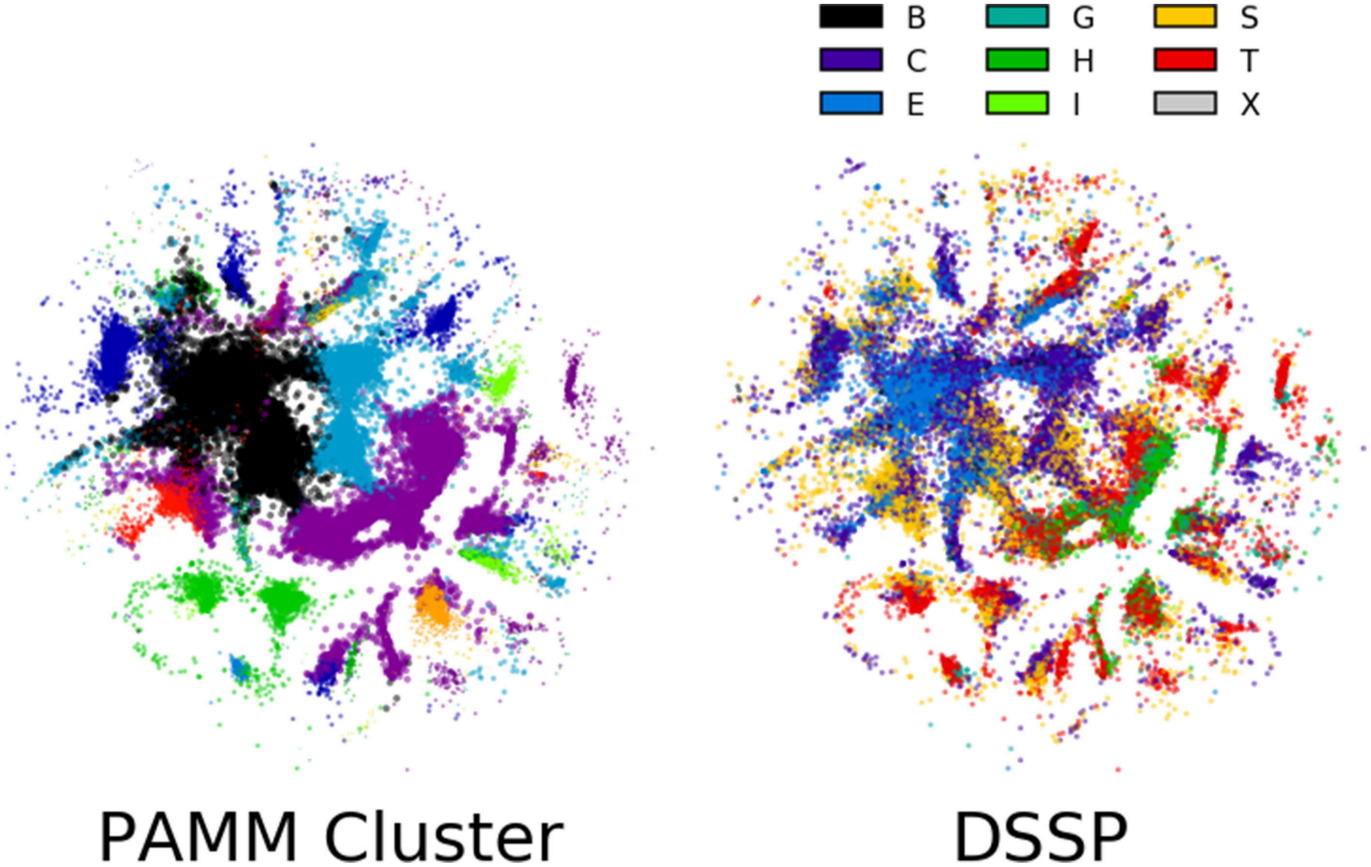
Sketch-map representations of 100,000 randomly selected points in the six-dimensional ϕ, ψ space. Each point is colored according to its PAMM cluster assignment and middle residue DSSP secondary structure assignment. The lack of clear grouping observed among secondary structures suggests that secondary structure cannot be assigned based on dihedral angles alone. The points that are colored by their PAMM cluster are also sized based on the cluster weight; points belonging to a cluster with higher weight are larger.

The sensitivity of the classification to the parameters of the method is a general issue with unsupervised schemes, for which it is difficult to define a quantitative measure of the quality of the classification, based on which the performance of the algorithm can be automatically optimized. One possible solution would be to couple the unsupervised classification to a supervised learning task, as we discuss below. Another possibility involves the direct inspection of the cluster structure, which requires, in the case of high-dimensional data, the application of another class of unsupervised learning algorithms that is aimed at obtaining a simplified low-dimensional representation. To this end, we have applied in Figure 7 the Sketch-map dimensionality reduction method (Ceriotti et al., 2011; Tribello et al., 2012; Ceriotti et al., 2013) to the six-dimensional dihedral data.

The guiding principle of Sketch-map is to project high-dimensional data into a lower dimension such that points that are close to one another in the high-dimensional space are also close to one another in reduced dimension, and similarly for points that are far apart. Each point in the Sketch-map projection of the six-dimensional ϕ, ψ space is colored by its PAMM cluster assignment and its DSSP secondary structure assignment (Figure 7; the Sketch-map projection colored by STRIDE secondary structure assignment is given in the Supplemental Material). The Sketch-map projection corroborates our earlier observations that, with the exception of the helices and strands, any given secondary structure is distributed widely across the high-dimensional space. However, one can observe that there is considerably less overlap between regions associated with different DSSP motifs, and it appears that the failure of recognizing these regions as separate clusters is more a consequence of the scattered distribution of points rather than a lack of resolving power.

### 3.3. SOAP Environments

**Figure 8:**
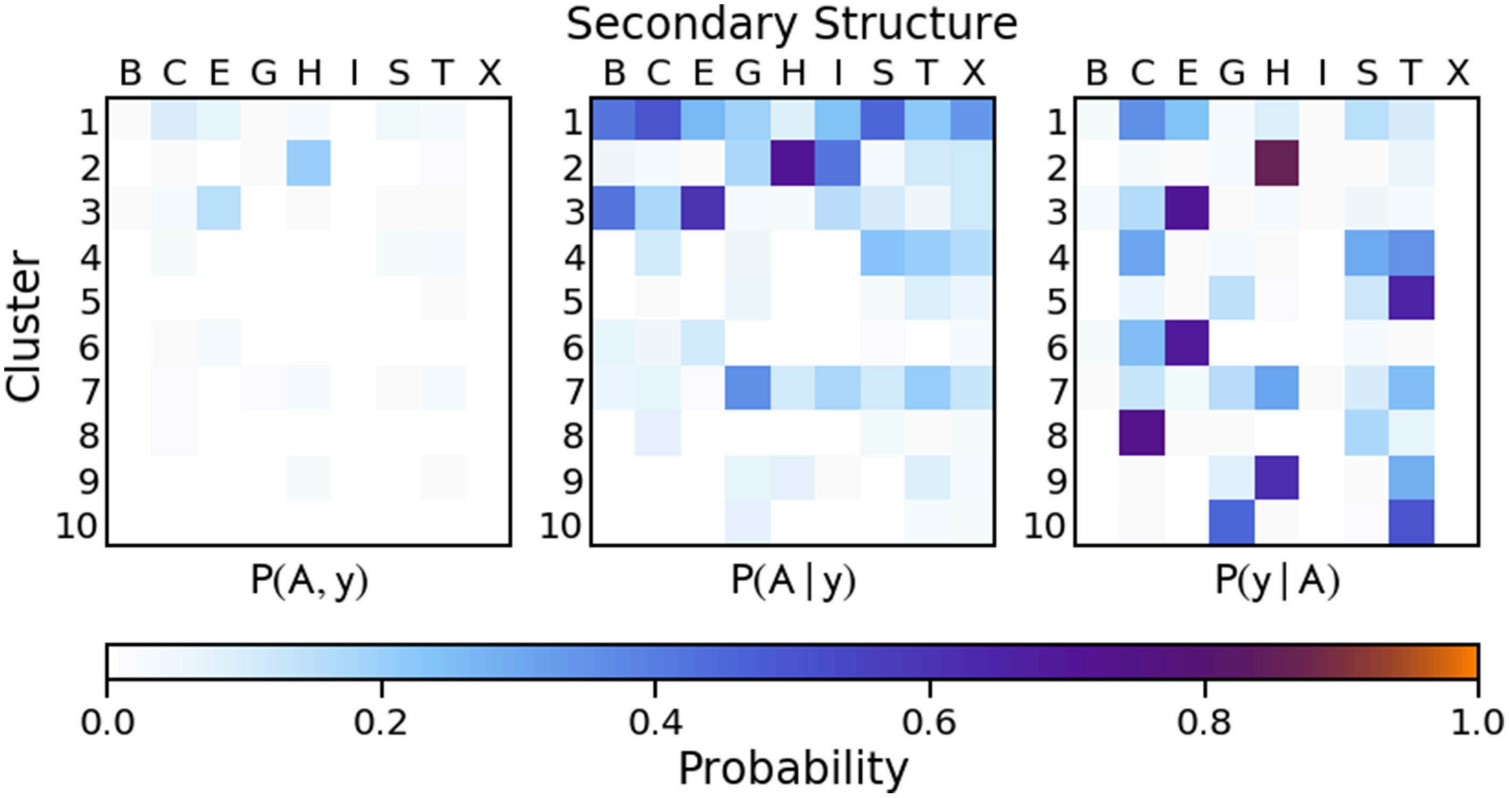
Joint and conditional probabilities for the PAMM clustering of the first two principal components of the reduced SOAP vectors describing each residue of the protein backbone, where *A* is the PAMM cluster assignment and *y* is the DSSP secondary structure classification.

While it appears that established secondary structure definitions are not associated with well-separated modes in the PDB data, we cannot exclude that this is due to an incomplete description, and that a structure representation encoding more information than the sequence of backbone dihedrals would show greater correspondence between data-driven motifs and established structural definitions. For this reason, we turn to a radically different approach to represent local motifs. We use a SOAP-based representation (whose details are discussed above and in the SI) of the protein backbone for comparison with established secondary structure definitions. A PAMM GMM based on reduced SOAP vectors forms the basis for a truly agnostic method for identifying structural motifs and classifying secondary structure in proteins, as the only required information is the positions of the atoms in the protein backbone. The joint and conditional probability distributions of the clusterized SOAP vectors and DSSP secondary structure assignment are given in Figure 8 (the probability distributions relative to the STRIDE assignment can be found in the Supplemental Material).

Compared to the dihedral angle probability distributions, the distributions based on a clustering of the SOAP vectors are more diffuse. Instead of the helices and strands being confined to one or two clusters as with the dihedral angles, in the SOAP clustering the helices and strands are divided among several clusters. However, from the perspective of the Q3 score, the SOAP representation performs as well as the dihedral angle representations, with scores in the range of 0.70–0.74 for two-, six- and ten-dimensional representations based on the principal components of the SOAP vectors.

### 3.4. Supervised Classification

The fact that increasing the complexity of the environment descriptors does not improve the match between PAMM PMIs and conventional secondary structure motifs suggests that the discrepancy is not due to lack of descriptive power, but to the fact that conventional motifs are not reflected in the environment distributions observed in the PDB. To substantiate this observation, we also use the dihedral angle and SOAP PCA representations to train an SVM to perform multiclass classification for the purpose of predicting secondary structures. The Q3 and Q8 scores resulting from SVMs built on the reduced SOAP representation and the dihedral angle representation at various dimensionalities are given in Table 3 and are seen to improve systematically when the dimensionality of the representation is increased—contrary to what observed with a PAMM analysis.

The improving Q3 and Q8 scores for the dihedral angles and reduced SOAP representations in the SVM coupled with the lack of obvious improvement in the cluster-based Q scores confirms that the limiting factor in the association between motifs is intrinsic to unsupervised learning. The reference heuristics—the DSSP and STRIDE secondary structure definitions—are simply not well-represented in the probability distribution of the data in the feature space that we use.

This simple example highlights both the difference in unsupervised and supervised learning methods while also emphasizing the importance of the choice of feature representation. A supervised learning scheme is well-suited to adapt an existing motif definition to a different representation of atomic environments, and—in the limit of a sufficiently large train set—serves as proof of whether the chosen representation is sufficiently complete to achieve an accurate classification. An unsupervised clustering model, on the other hand, is useful for finding new patterns in feature space. Provided that the representation is complete, it also can serve as validation for established pattern recognition heuristics, showing whether the presence of well separate motifs is robust to the choice of structural representation.

By comparing chains of dihedrals and backbone SOAP principal components, we have shown that the two representations possess a similar resolving power for a given size, and yield SOAP motifs that compare roughly in the same way to the DSSP/STRIDE classifications of secondary structure. While dihedral angles are certainly simpler and more straightforward to incorporate into existing analysis schemes, the general-purpose nature of SOAP makes the latter more suitable to be extended to different classes of supramolecular structures, and provides a somewhat less biased starting point for subsequent machine learning analyses.

## 4. Conclusions

In this work we have applied data-driven analysis techniques to experimental atomistic structure data of polypeptides extracted from the Protein Data Bank. Our objective has been to demonstrate that a generally applicable analysis protocol, that relies on little specific information for the system at hand can be used to re-discover some of the fundamental atomic-scale motifs that underlie the formation of complex supramolecular structures—specifically the hydrogen bond and secondary structure patterns. For this purpose, we used PAMM, a density-based algorithm that recognizes and associates local maxima in atomic feature space with particularly stable, frequently occurring configurations to highlight some of the shortcomings of more traditional definitions. For instance, we showed how conventional bond–angle criteria to recognize hydrogen bonds rely on multiple additional heuristics to avoid incorrectly classifying other recurring motifs that are associated to covalently bound groups. Furthermore, we quantified the substantial differences between various hydrogen-bond “flavors,” underscoring the advantages of an adaptive, automatic definition.

The case of secondary structure patterns gave us the opportunity to compare the use of conventional representations of local atomic structure (backbone dihedrals) with an even more generally applicable strategy based on the principal components of the SOAP power spectrum. Despite being very different in spirit, the two representations yield very similar results; there is a good match between PAMM-based patterns and traditional heuristics for what concerns helices and strands, but rather poor agreement for other, less common motifs. By comparing representations of different complexity, and the outcome of both supervised and unsupervised classification schemes, we have shown that the conventional secondary structure recognition methods reflect only in part the intrinsic distribution of data of protein structures in the PDB.

While conventional secondary structure motifs have the advantage of being linked to structure–property relations and important design principles and have survived the test of time, data-driven definitions such as PAMM-based PMIs can be more easily adapted to specific simulations or, as in the present case, experimental data sets. Their robustness is highlighted by clustering outcomes that are rather insensitive to the choice of the structure representation. The possibility of using generic representations, such as the list of backbone dihedrals, or even more abstract feature vectors such as the SOAP power spectrum, makes a PAMM analysis well-suited for application to different classes of supramolecular and self-assembly problems, where less prior knowledge is available to define heuristic criteria. Finally, given that PMIs are smooth, differentiable functions that depend exclusively on atom coordinates, they show great promise for use in combination with automatic collective variable determination and in accelerated sampling schemes to probe structural transitions and rare events.

## Author Contributions

BH performed simulations, analyzed them, wrote pre- and post- processing code, and prepared figures. PG ran preliminary tests and implemented the PAMM analysis software. All authors contributed to the design of the simulations and the writing of the text.

### Conflict of Interest Statement

The authors declare that the research was conducted in the absence of any commercial or financial relationships that could be construed as a potential conflict of interest.
