# Supplementary material for: Atomic Motif Recognition in (Bio)Polymers: Benchmarks From the Protein Data Bank
Source: Front Mol Biosci. 2019 Apr 18;6:24. doi: 10.3389/fmolb.2019.00024 (PMC6482324; doi:10.3389/fmolb.2019.00024)
Supplement: Supplementary file 1 [file Data_Sheet_1.pdf]

Atomic Motif Recognition in (Bio)Polymers: Benchmarks from the Protein Data Bank

Supplementary Information

Benjamin A. Helfrecht, Piero Gasparotto, Federico Giberti, and Michele Ceriotti*

Laboratory of Computational Science and Modeling, Institute of Materials, École Polytechnique Fédérale de Lausanne, 1015 Lausanne, Switzerland

E-mail: michele.ceriotti@epfl.ch

1 Dataset Statistics

1.1 Hydrogen Bonding

Applying the dataset refinements stated in the Methods of the main text yielded 872 crystal structures, from which 418,865 N−H···N triplets, 918,014 N−H···O triplets, 42,650 O−H···O triplets, and 57,572 O−H···N triplets were extracted and used to build the Gaussian mixture models. The DSSP hydrogen bonding dataset was based on the same 872 protein crystal structures, but only 844 structures contained valid (N−H, C=O) pairs according to the criteria outlined in the Methods. Hence, the DSSP hydrogen bonding dataset included 552,281 potential N−H···O hydrogen bonds. Water and small molecules were excluded; nonstandard amino acids were included.

1.2 Dihedral Angles

Our dataset contains 12,713 protein crystal structures obtained by X-ray diffraction with a resolution better than 1.5 Å. Backbone dihedral angles could only be extracted from 12,708 structures, totaling 4,275,677 residues. Water and small molecules were excluded; nonstandard amino acids were included. DSSP secondary structure assignments are from version 2.2.1 of the software. No version information was available for STRIDE.

1.3 SOAP Representation

The same 12,708 structures from the dihedral angle dataset were used in the analysis based on the SOAP representation. However, the atomic positions of the proline of residue 2 in chain E of structure 3ADM are identical to the atomic positions of residue 5 of the same chain. Overlapping atomic positions causes the SOAP representation to fail, and so residue 2 of chain E in structue 3ADM was discarded (in addition to the nitrogen of residue 3, which has identical coordinates to the nitrogen of residue 6). Therefore, 4,275,676 residues were included in our SOAP analysis. These residues—common to both the dihedral angle and SOAP datasets—were used for the support vector machine computations of Q3 and Q8 scores.

2 SOAP Parameters

In addition to the setup explained in the Methods of the main text, our SOAP representation employed the following parameters: `n_max` = 12, `l_max` = 9, `cutoff` = 6.0 Å, `cutoff_transition_width` = 0.5, `atom_sigma` = 0.5. All other parameters were set to the default values. Explanations of the parameters are given in the `quippy` library reference, available at `https://libatoms.github.io/QUIP/descriptors.html`.

3 Sketch-map Parameters

The six-dimensional Sketch-map of backbone dihedral angles was constructed using 500 landmark points, $\sigma = 2.5$, $A = B = 4$, and $a = b = 2$. See Ref. S1 for details on the significance of the parameters and guidelines for their selection.

4 Hydrogen Bonding PMIs

For reference, the DSSP hydrogen bond definition is as follows:[S2]

$$E = q_1 q_2 f \left(\frac{1}{d_{ON}} + \frac{1}{d_{CH}} - \frac{1}{d_{OH}} - \frac{1}{d_{CN}} \right), \tag{S1}$$

which is based on the distances d between the atoms participating in the $\mathrm{C} = \mathrm{O}$ bond of one residue and the $\mathrm{N} - \mathrm{H}$ bond of another residue. The factor $f = 332$ gives the energy E in kcal/mol with d in angstroms and e as the unit electron charge. Configurations with $E < -0.5$ kcal/mol are considered by DSSP to be hydrogen bonds.

The probability distribution of acceptor–hydrogen and donor–acceptor distances of the 1,437,101 donor–hydrogen–acceptor triplets across all four hydrogen bond flavors is shown in Fig. S1.

A comparison of the PAMM PMIs of the four hydrogen bond flavors with a distance–angle hydrogen bond definition is given in Fig. S2, highlighting the greater selectivity of the GMM-based definition when the geometric definition is applied blindly. The PAMM PMIs of the different hydrogen bond flavors are compared in Fig. S3, which shows that hydrogen bonding geometries can vary substantially depending on the species of the donor and acceptor.

A PAMM clustering of backbone-only N−H···N and N−H···O triplets is shown in Fig. S4. No hydrogen bond cluster is evident in the N−H···N case, suggesting that the N−H···N hydrogen bonds we observe occur almost exclusively between amino acid side chains. Similarly, the shape and location of the backbone-only N−H···O hydrogen bond PMI is very similar

to that of the total N−H···O hydrogen bond PMI, suggesting that the N−H···O hydrogen bonds are predominantly those existing in the protein backbone.

Note that the distribution for N−H···O configurations in the backbone (Fig. S4) is different from the distribution in the DSSP definition (Fig. 2 in the main text), which also considers backbone N−H···O geometries. This is because the DSSP definition applies additional constraints on the types of "acceptable" geometries, namely that the N and O atoms must be in different residues and that the H atom must be bound to the N atom.

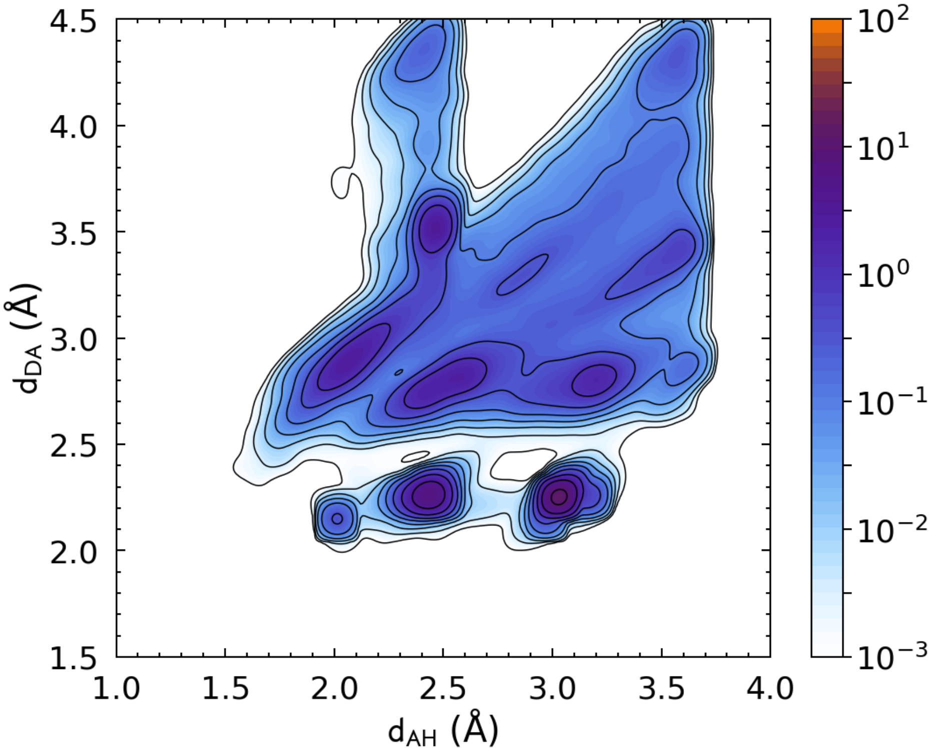

Figure S1: Total probability distribution of d_{AH} and d_{DA} across all hydrogen bond flavors. The distribution is peaked strongly at (d_{AH} = 3.0, d_{DA} = 2.25) as a result of common $\mathrm{N-H\cdots O}$ geometries in the protein backbone corresponding to N and O atoms in the same or directly adjacent residues. Contours are equally spaced on a logarithmic scale.

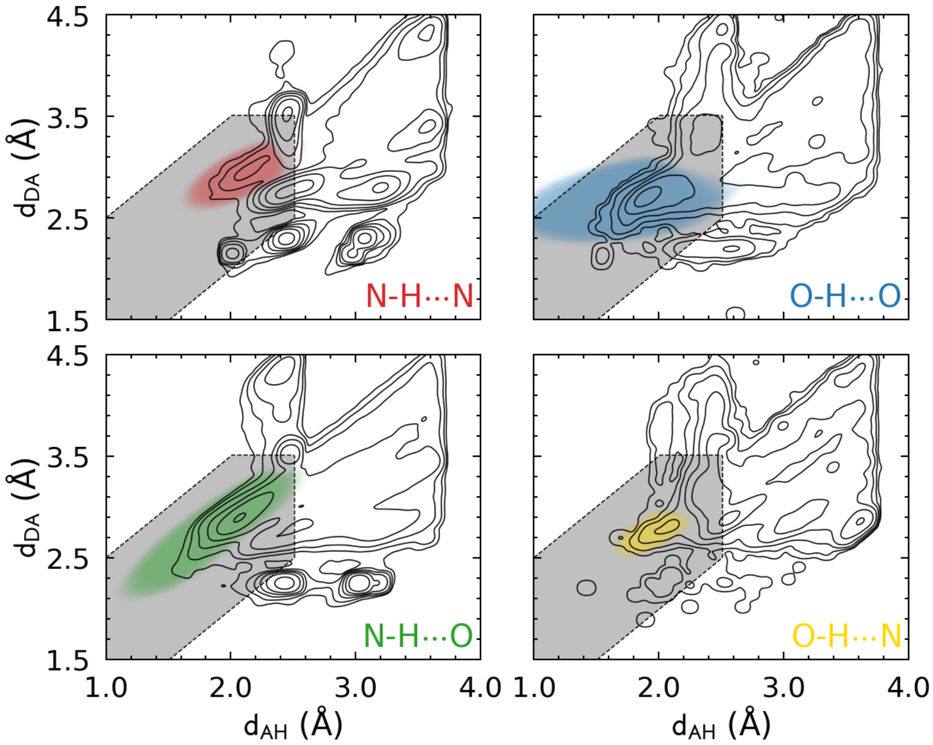

Figure S2: Comparison between the PAMM PMIs of the four hydrogen bond flavors and the distance–angle hydrogen bond definition superimposed on a histogram of the acceptor–hydrogen and donor–acceptor distances for the hydrogen bond flavor of interest. Contours are equally spaced on a logarithmic scale.

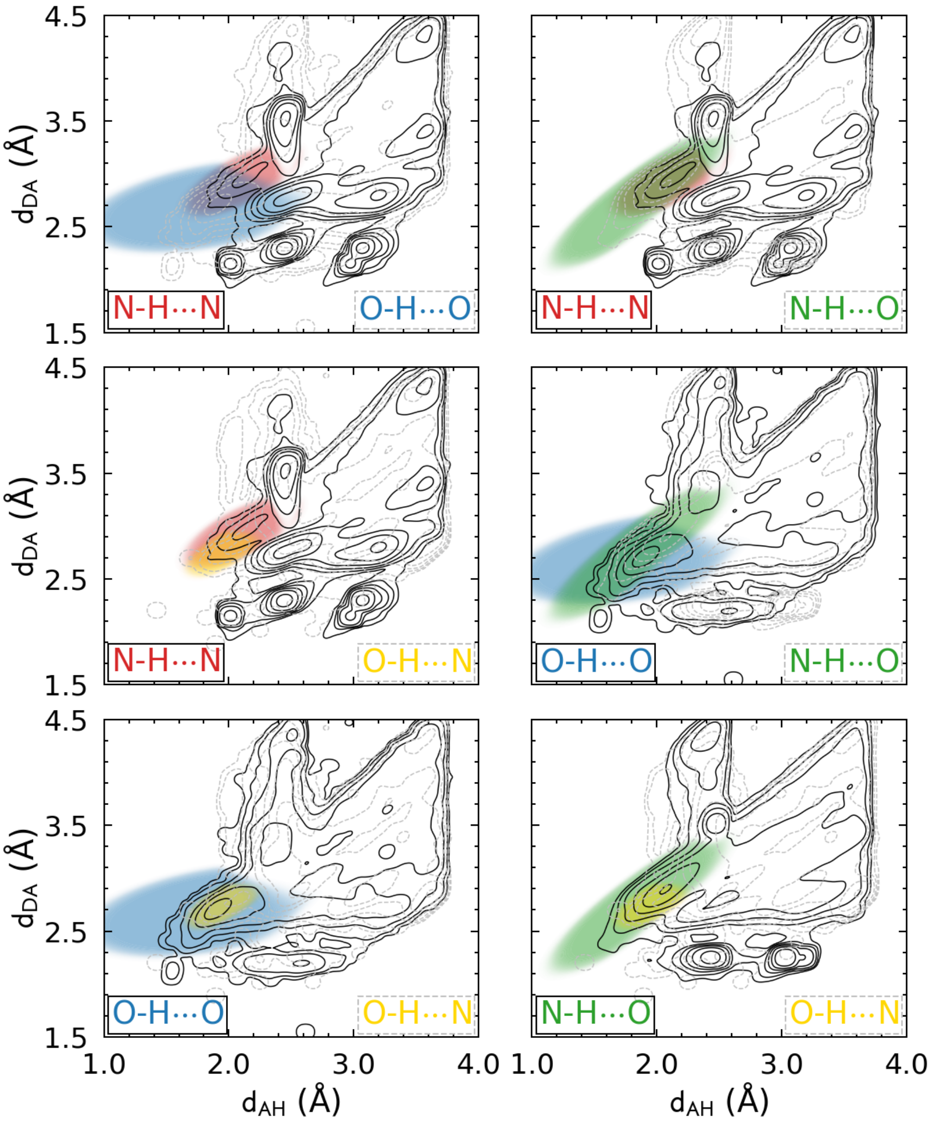

Figure S3: Comparison between the PAMM PMIs of the four different hydrogen bond flavors. The linestyle of the box enclosing the label of the hydrogen bond flavor corresponds to the linestyle of the log-spaced contours of the underlying (d_{AH}, d_{DA}) distribution for that hydrogen bond flavor.

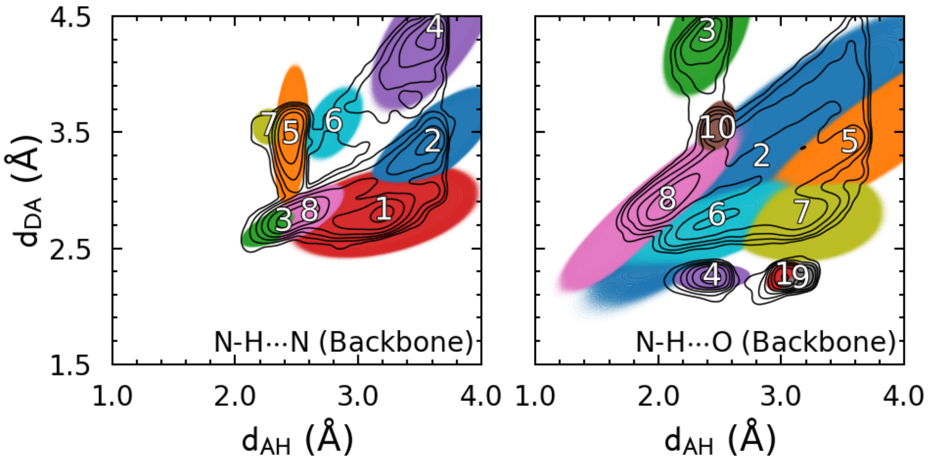

Figure S4: PAMM clustering for N−H···O and N−H···N with a background parameter $\zeta = 10^{-5}$, where the donor and acceptor atoms are a part of the protein backbone only.

5 Probability Distributions

5.1 Agreement between DSSP and STRIDE

In general, DSSP and STRIDE agree quite well in assigning secondary structures, as shown in Fig. S5. The main difference between the two methods is in the classification of π-helices (and the fact that only DSSP assigns the label “bend”). In particular, DSSP classifies many more residues as π-helices than does STRIDE; if STRIDE assigns a particular residue to be a π-helix, there is a sizeable probability that DSSP will also classify that same residue as a π-helix, but most of the DSSP π-helices are assigned as α-helices by STRIDE. Similarly, most of the residues classified as a bend by DSSP are assigned to the turn or loop/coil categories by STRIDE, as STRIDE does not explicitly include bends as a secondary structure type.

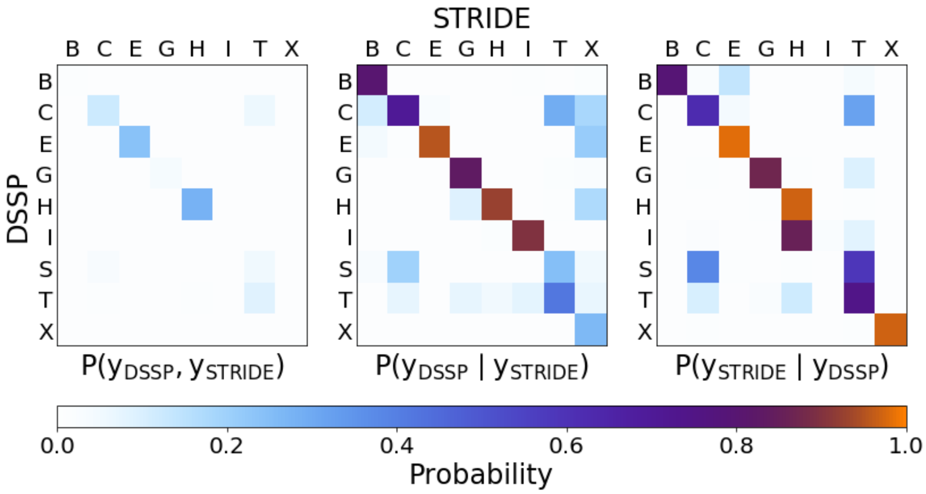

Figure S5: Joint and conditional probabilities for the secondary structures obtained from DSSP and STRIDE, where y_{DSSP} and y_{STRIDE} are the secondary structure assignments from DSSP and STRIDE, respectively.

5.2 Dihedral Angles

Fig. S6 is the STRIDE analog to the DSSP dihedral angle probability distribution presented in the main text. Figs. S7–S10 are the DSSP and STRIDE probability distributions in six and ten dimensions. The higher dimensional dihedral angle spaces are formed by considering the dihedrals from consecutive residues. In all cases, the helices and strands are represented primarily by one or two clusters, while the other secondary structures tend to be spread across several clusters.

Fig. S11 shows contour plots of 100,000 randomly selected dihedral angle pairs separated according to their STRIDE-assigned secondary structure. Fig. S12 shows the Sketch-map representation of 100,000 randomly selected points for the six-dimensional dihedral angle representation (formed by considering a sequence of the ϕ and ψ angles in three consecutive residues and the STRIDE assignment of the middle residue). The corresponding figures for DSSP assignments are given in the main text.

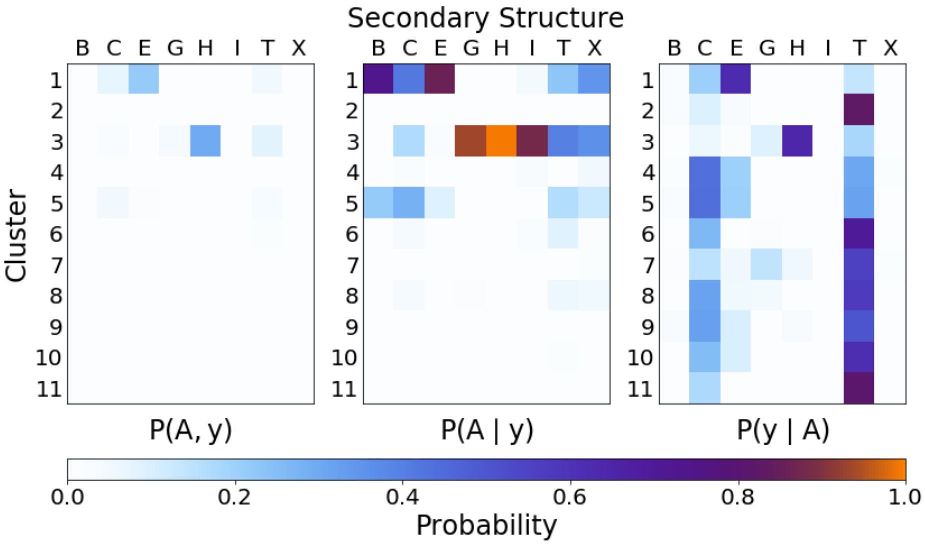

Figure S6: Joint and conditional probabilities for the secondary structures obtained from STRIDE and the clustering of dihedral angles from PAMM, where A is the cluster assignment and y the secondary structure classification.

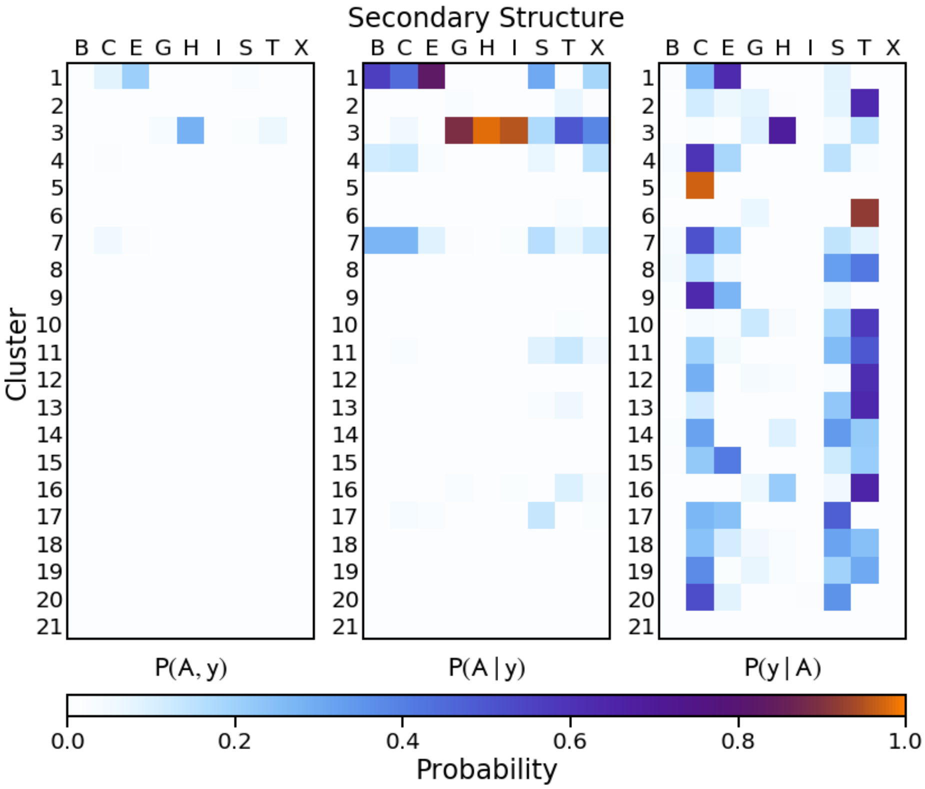

Figure S7: Joint and conditional probabilities for the clustering of dihedral angles from PAMM for three consecutive residues (a six-dimensional ϕ, ψ space), where A is the PAMM cluster assignment and y is the DSSP secondary structure assignment of the middle residue.

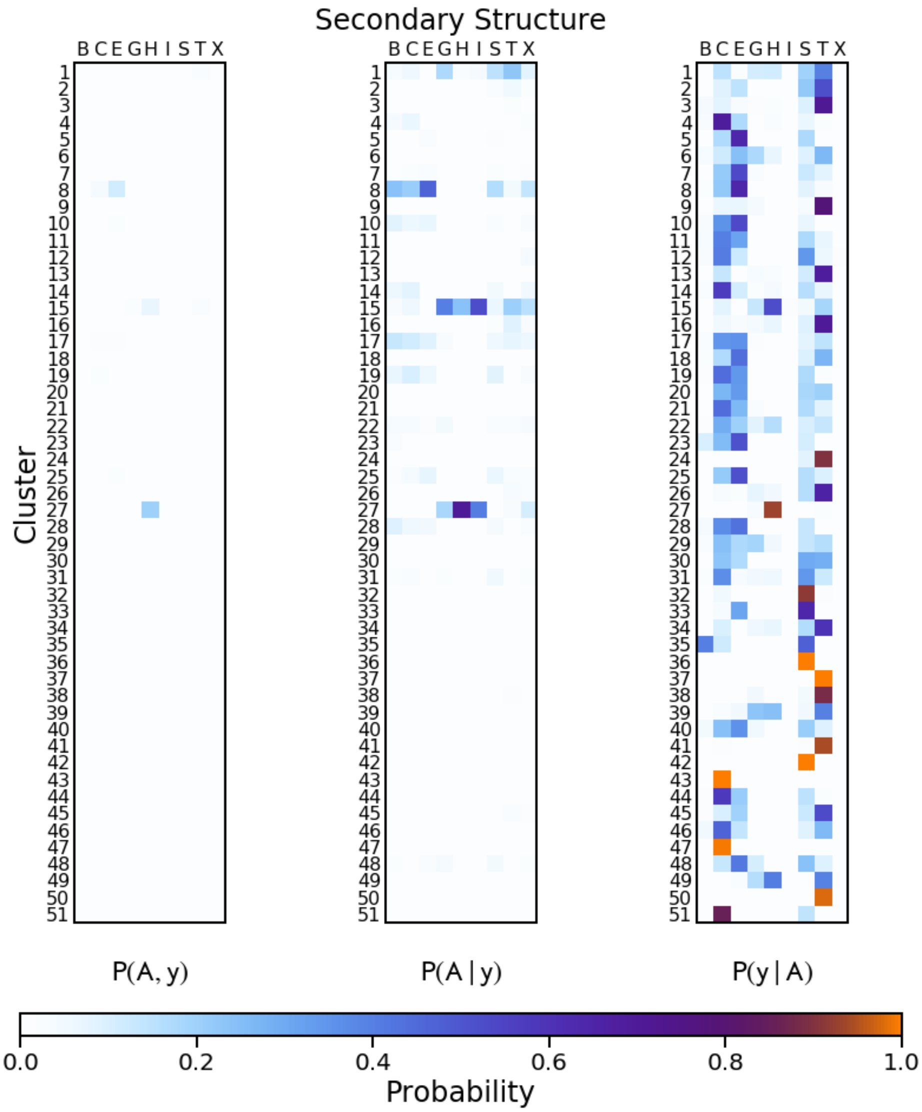

Figure S8: Joint and conditional probabilities for the clustering of dihedral angles from PAMM for five consecutive residues (a ten-dimensional ϕ, ψ space), where A is the PAMM cluster assignment and y is the DSSP secondary structure assignment of the middle residue.

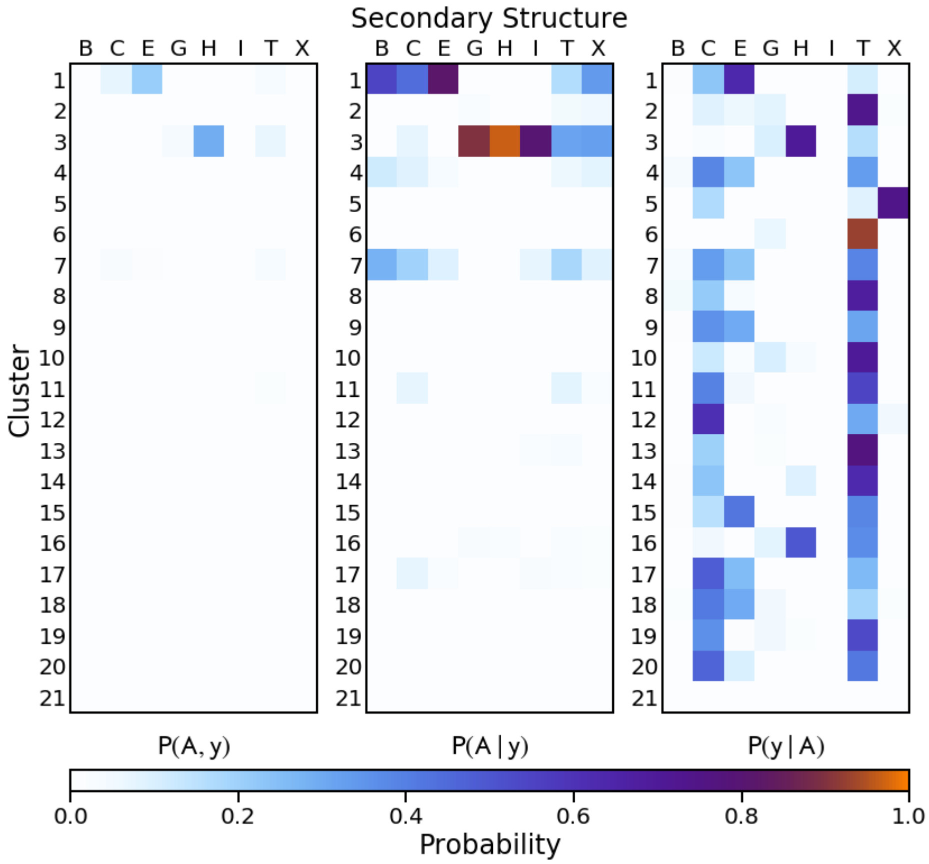

Figure S9: Joint and conditional probabilities for the clustering of dihedral angles from PAMM for three consecutive residues (a six-dimensional ϕ, ψ space), where A is the PAMM cluster assignment and y is the STRIDE secondary structure assignment for the middle residue.

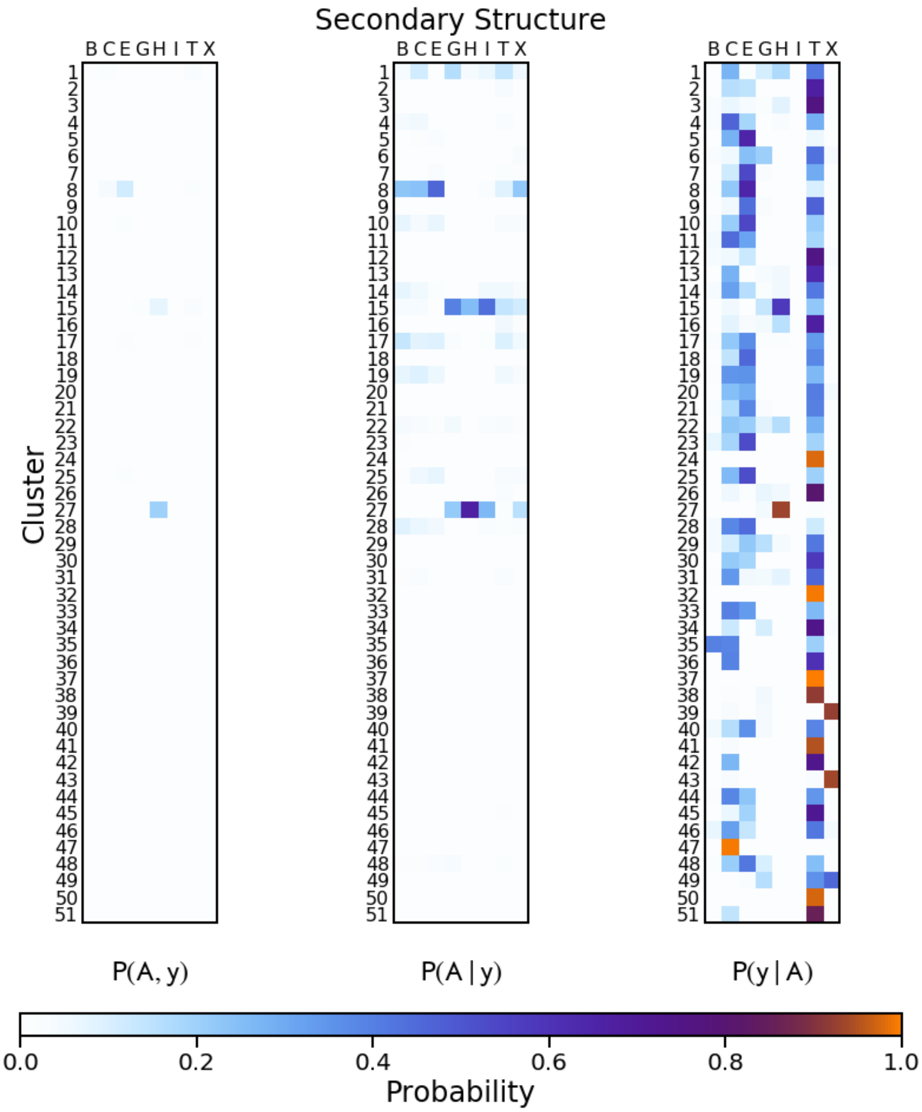

Figure S10: Joint and conditional probabilities for the clustering of dihedral angles from PAMM for five consecutive residues (a ten-dimensional ϕ, ψ space), where A is the PAMM cluster assignment and y is the STRIDE secondary structure assignment of the middle residue.

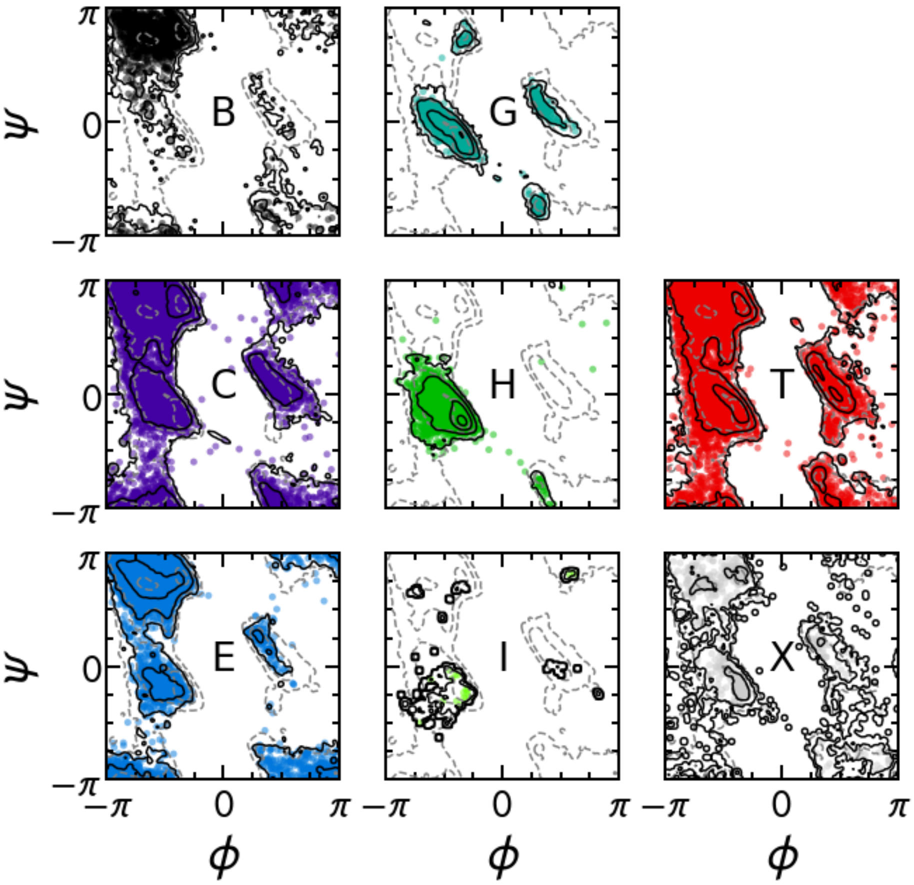

Figure S11: Collection of 100,000 randomly selected (ϕ, ψ) pairs, separated according to the STRIDE secondary structure classification of each pair. Solid contours correspond to the distribution of the secondary structure of interest; dashed contours correspond to the total distribution of all ϕ, ψ angles. Contours are equally spaced on a logarithmic scale.

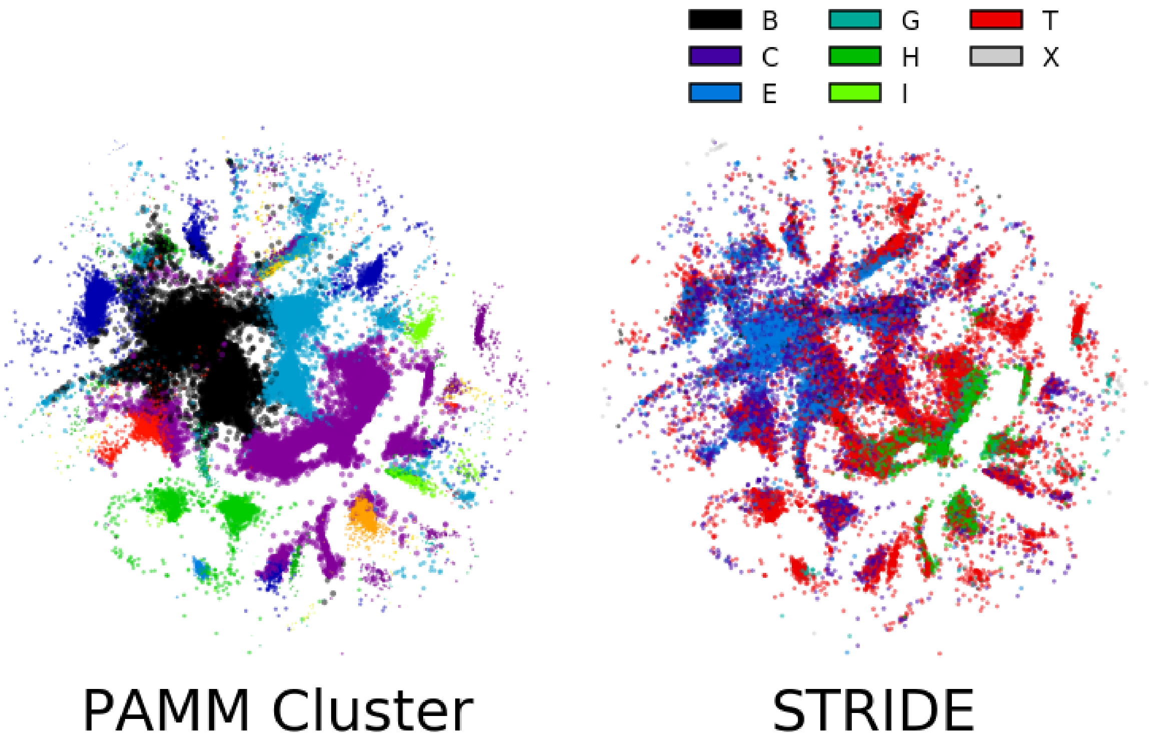

Figure S12: Sketch-map representations of 100,000 randomly selected points in the six-dimensional ϕ, ψ space. Each point is colored according to its PAMM cluster assignment and middle residue STRIDE secondary structure assignment. The lack of clear grouping observed among secondary structures suggests that secondary structure cannot be assigned based on dihedral angles alone. The points that are colored by their PAMM cluster are also sized based on the cluster weight; points belonging to a cluster with higher weight are larger.

5.3 SOAP

Figs. S13 and S14 show the distributions of the 2D SOAP PCA data separated by secondary structure label, analogous to Fig. S11 and Fig. 5 in the main text. Small point sizes are used here to better show the peaks in the distribution; as a result the more sparsely populated secondary structures become washed out. Fig. S15 is the STRIDE analog to the DSSP SOAP probability distribution presented in the main text. Figs. S16–S19 are the DSSP and STRIDE probability distributions in six and ten dimensions. The higher dimensional SOAP spaces are formed by considering additional principal components of the collection of SOAP vectors after reducing the number of components via farthest point selection. In contrast to

the dihedral angle representations, clustering based on the SOAP representation does not result in the strands and helices being clearly confined to one or two clusters, particularly in the two- and six-dimensional cases.

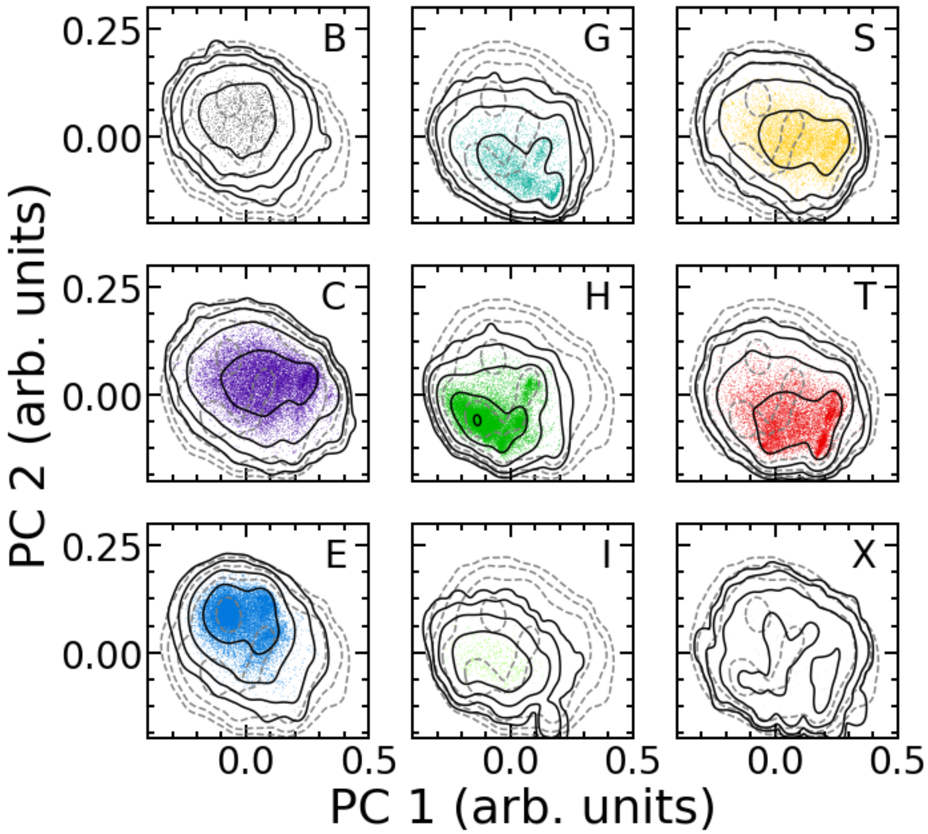

Figure S13: Collection of 100,000 randomly selected SOAP vectors projected onto the first two principal components of the dataset and separated according to the DSSP secondary structure classification of each point. Solid contours correspond to the distribution of the SOAP vectors with the secondary structure label of interest; dashed contours correspond to the total distribution of all vectors in the 2D SOAP PCA space. Contours are equally spaced on a logarithmic scale.

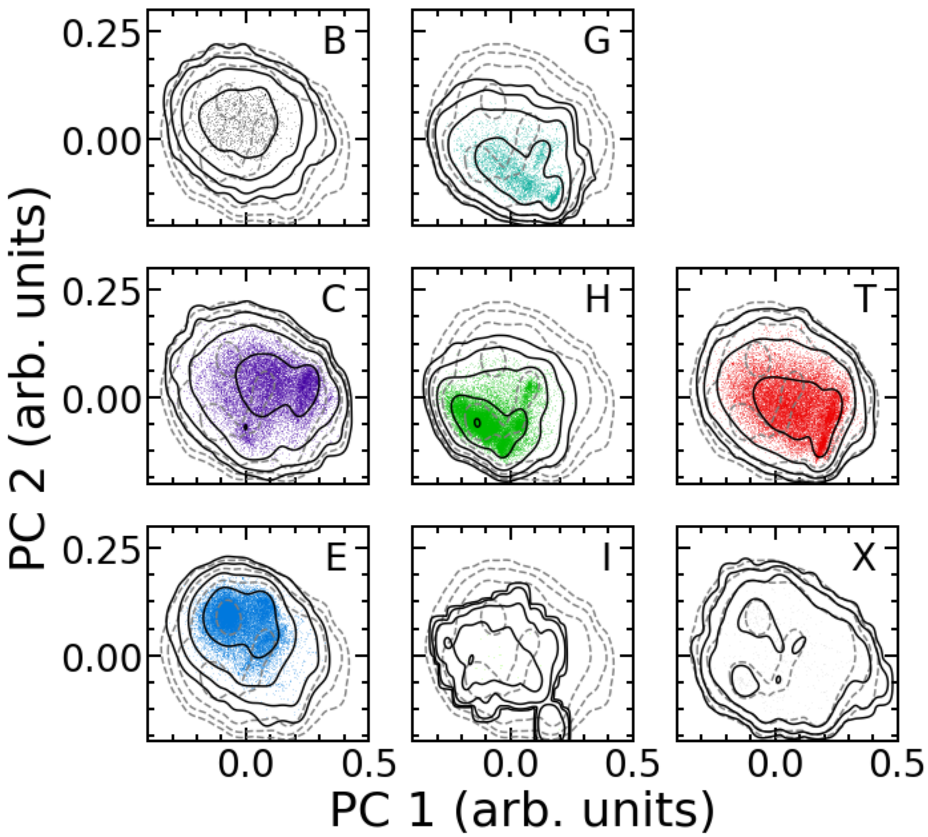

Figure S14: Collection of 100,000 randomly selected SOAP vectors projected onto the first two principal components of the dataset and separated according to the STRIDE secondary structure classification of each point. Solid contours correspond to the distribution of the SOAP vectors with the secondary structure label of interest; dashed contours correspond to the total distribution of all vectors in the 2D SOAP PCA space. Contours are equally spaced on a logarithmic scale.

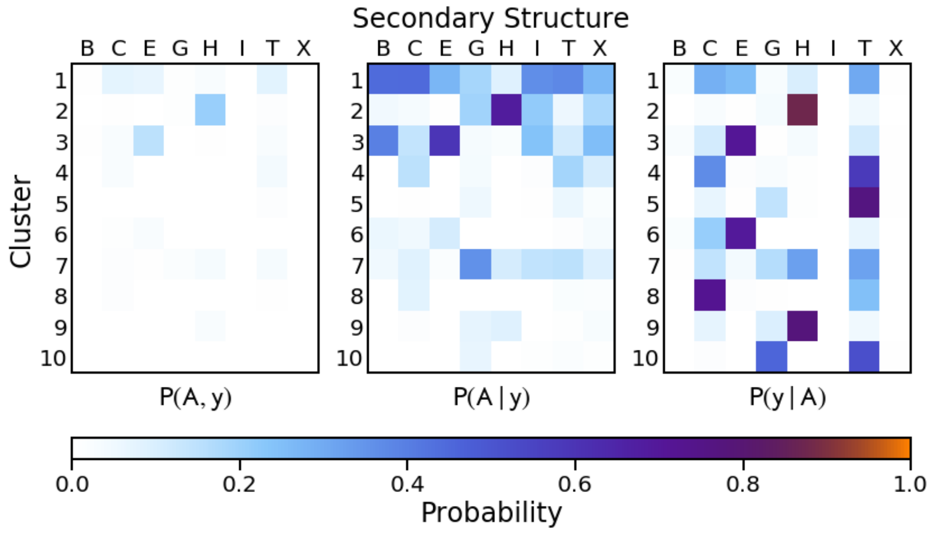

Figure S15: Joint and conditional probabilities for the PAMM clustering of the first two principal components of the reduced SOAP vectors describing each residue of the protein backbone, where A is the PAMM cluster assignment and y is the STRIDE secondary structure classification.

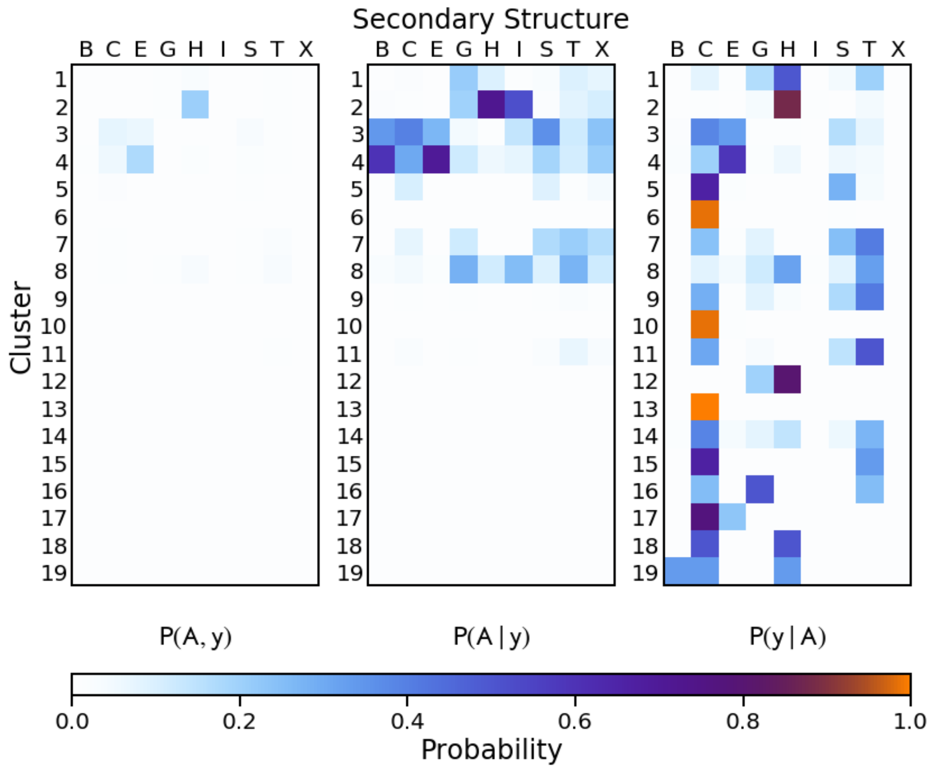

Figure S16: Joint and conditional probabilities for the PAMM clustering of the first six principal components of the reduced SOAP vectors describing each residue of the protein backbone, where A is the PAMM cluster assignment and y is the DSSP secondary structure classification.

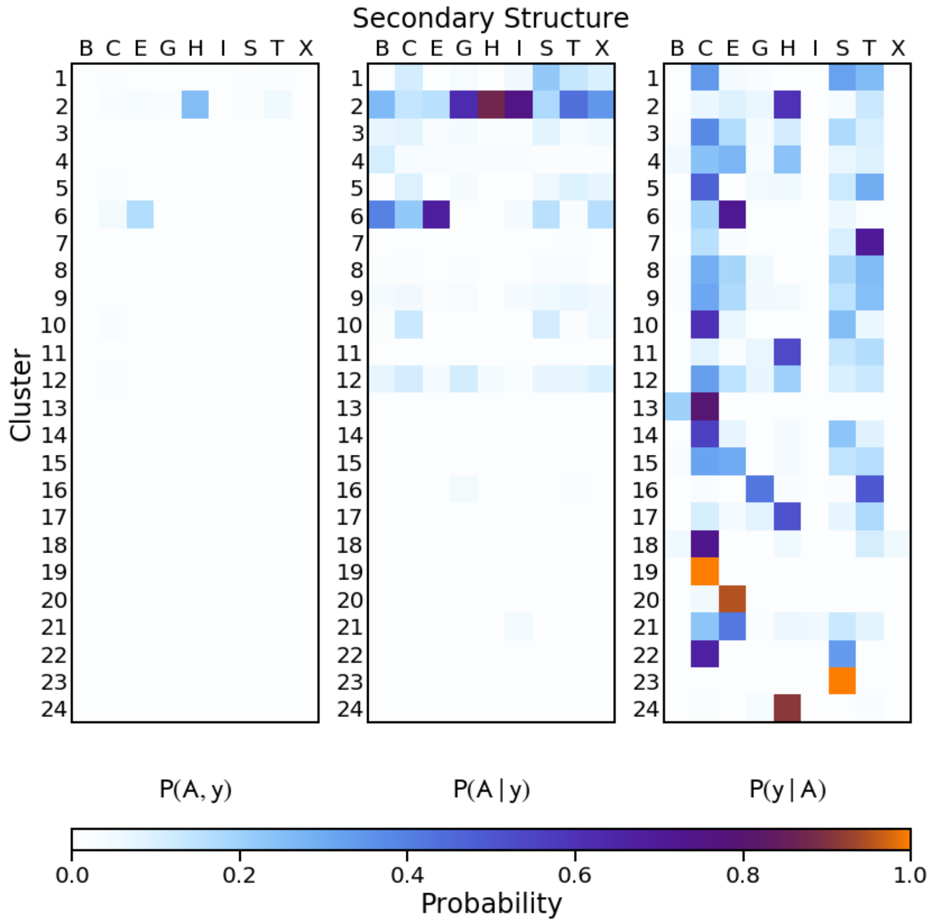

Figure S17: Joint and conditional probabilities for the PAMM clustering of the first ten principal components of the reduced SOAP vectors describing each residue of the protein backbone, where A is the PAMM cluster assignment and y is the DSSP secondary structure classification.

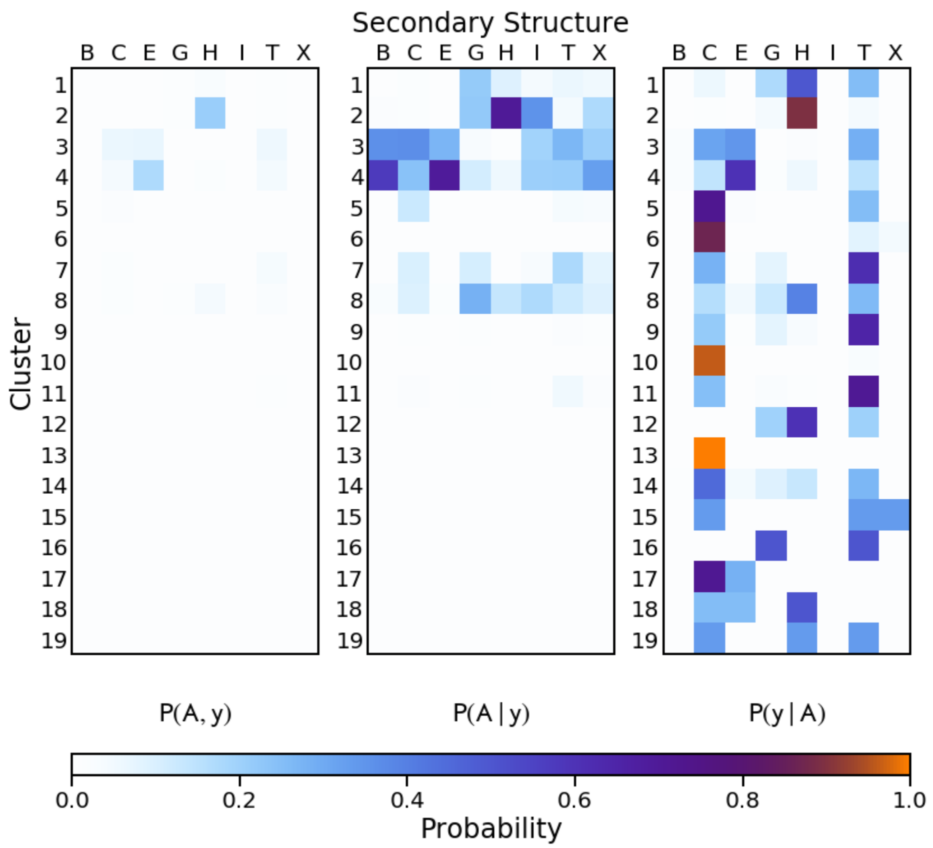

Figure S18: Joint and conditional probabilities for the PAMM clustering of the first six principal components of the reduced SOAP vectors describing each residue of the protein backbone, where A is the PAMM cluster assignment and y is the STRIDE secondary structure classification.

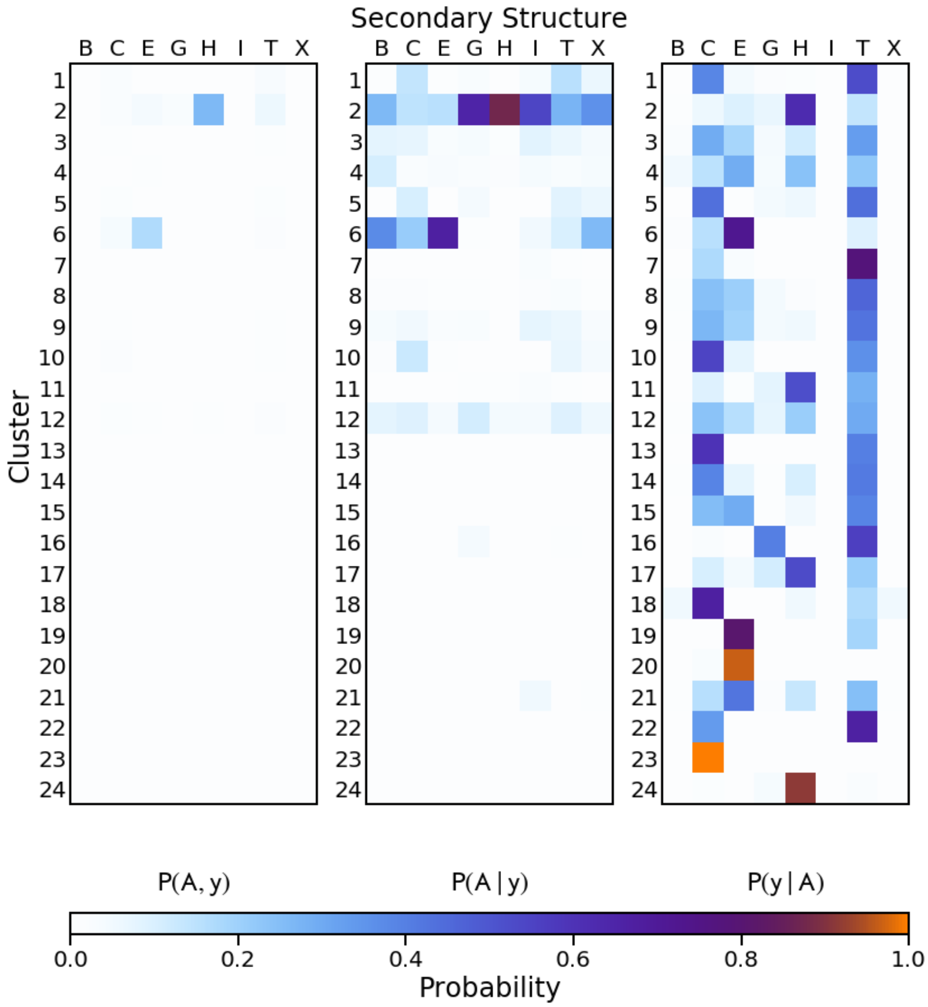

Figure S19: Joint and conditional probabilities for the PAMM clustering of the first ten principal components of the reduced SOAP vectors describing each residue of the protein backbone, where A is the PAMM cluster assignment and y is the STRIDE secondary structure classification.

6 Supervised Learning

Figures S20 and S21 show the learning curves of the Q3 and Q8 scores relative to DSSP and STRIDE for the multiclass support vector machine (SVM). Learning saturates more quickly for the descriptors of lower dimensionality. Each point in each curve is an average score over five separate constructions of the SVM, each time using a new random subset of 200,000 residues.

Table S1 provides the computed Q3 and Q8 scores for the dihedral angle and SOAP representations associated with the STRIDE secondary structure classification, similar to Table 3 in the main text that uses the DSSP assignments.

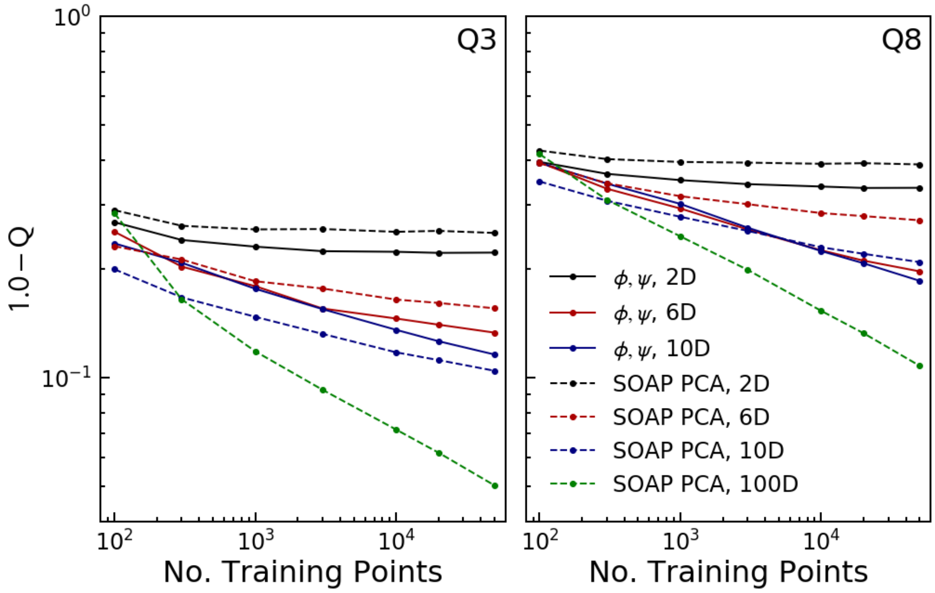

Figure S20: Learning curves of Q3 and Q8 scores relative to DSSP for the multiclass SVM based on backbone dihedral angles and a PCA of the SOAP representation with various degrees of information content (i.e., the dimensonality of the descriptor).

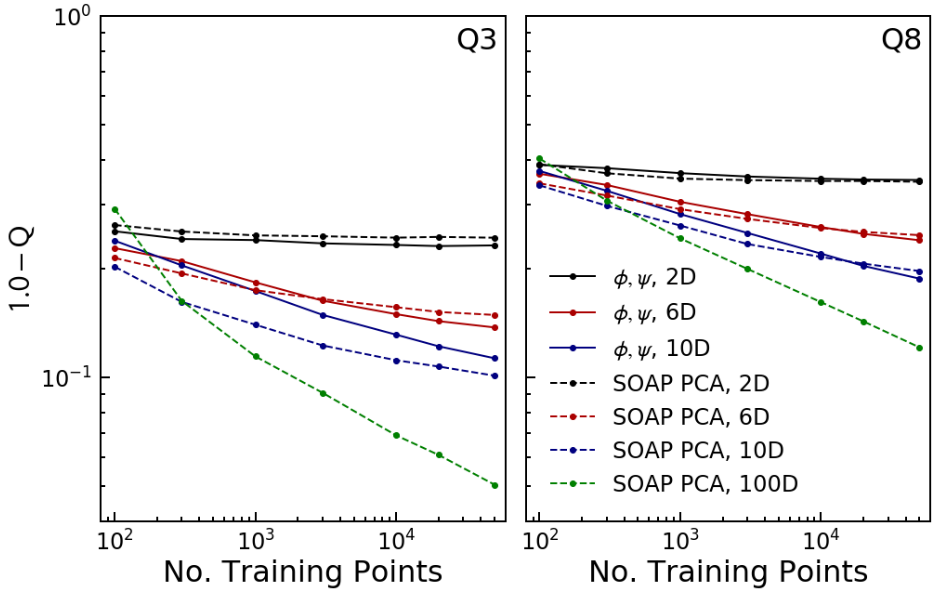

Figure S21: Learning curves of Q3 and Q8 scores relative to STRIDE for the multiclass SVM based on backbone dihedral angles and a PCA of the SOAP representation with various degrees of information content (i.e., the dimensonality of the descriptor).

Table S1: Q3 and Q8 scores relative to STRIDE for PAMM PMI and SVM predictions of secondary structure based on a PCA of SOAP vectors and dihedral angles at various dimensionality. The reported SVM scores are an average over five separate constructions of the SVM, each time using a new random subset of 200,000 residues, with 50,000 of these serving as the training set.

	PAMM PMI		SVM	
Representation	Q3	Q8	Q3	Q8
ϕ, ψ (2D)	0.72	0.61	0.77	0.65
ϕ, ψ (6D)	0.74	0.62	0.86	0.76
ϕ, ψ (10D)	0.73	0.62	0.89	0.81
SOAP PCA (2D)	0.74	0.60	0.76	0.65
SOAP PCA (6D)	0.72	0.60	0.85	0.75
SOAP PCA (10D)	0.71	0.58	0.90	0.80
SOAP PCA (100D)	—	—	0.95	0.88

References

[S1] Ceriotti, M.; Tribello, G. A.; Parrinello, M. Demonstrating the Transferability and the Descriptive Power of Sketch-Map. *Journal of Chemical Theory and Computation* **2013**, *9*, 1521–1532.

[S2] Kabsch, W.; Sander, C. Dictionary of Protein Secondary Structure: Pattern Recognition of Hydrogen-Bonded and Geometrical Features. *Biopolymers* **1983**, *22*, 2577–2637.
